# ICOS signaling promotes a secondary humoral response after re-challenge with *Plasmodium chabaudi chabaudi* AS

**DOI:** 10.1371/journal.ppat.1008527

**Published:** 2020-04-29

**Authors:** Leah E. Latham, Daniel J. Wikenheiser, Jason S. Stumhofer

**Affiliations:** University of Arkansas for Medical Sciences, Department of Microbiology and Immunology, Little Rock, AR, United States of America; University of Manchester, UNITED KINGDOM

## Abstract

The co-stimulatory molecule ICOS is associated with the induction and regulation of T helper cell responses, including the differentiation of follicular helper T (Tfh) cells and the formation and maintenance of memory T cells. However, the role of ICOS signaling in secondary immune responses is largely unexplored. Here we show that memory T cell formation and maintenance are influenced by persistent infection with *P*. *chabaudi chabaudi* AS infection, as memory T cell numbers decline in wild-type and *Icos*^*-/-*^ mice after drug-clearance. Following drug-clearance *Icos*^*-/-*^ mice display a relapsing parasitemia that occurs more frequently and with higher peaks compared to wild-type mice after re-challenge. The secondary immune response in *Icos*^*-/-*^ mice is characterized by significant impairment in the expansion of effector cells with a Tfh-like phenotype, which is associated with a diminished and delayed parasite-specific Ab response and the absence of germinal centers. Similarly, the administration of an anti-ICOSL antagonizing antibody to wild-type mice before and after reinfection with *P*. *c*. *chabaudi* AS leads to an early defect in Tfh cell expansion and parasite-specific antibody production, confirming a need for ICOS-ICOSL interactions to promote memory B cell responses. Furthermore, adoptive transfer of central memory T (T_CM_) cells from wild-type and *Icos*^*-/-*^ mice into *tcrb*^*-/-*^ mice to directly evaluate the ability of T_CM_ cells to give rise to Tfh cells revealed that T_CM_ cells from wild-type mice acquire a mixed Th1- and Tfh-like phenotype after *P*. *c*. *chabaudi* AS infection. While T_CM_ cells from *Icos*^*-/-*^ mice expand and display markers of activation to a similar degree as their WT counterparts, they displayed a reduced capacity to upregulate markers indicative of a Tfh cell phenotype, resulting in a diminished humoral response. Together these findings verify that ICOS signaling in memory T cells plays an integral role in promoting T cell effector responses during secondary infection with *P*. *c*. *chabaudi* AS.

## Author summary

Malaria, which is caused by the protozoan parasite *Plasmodium*, remains a major global health problem, as over 400,000 people die from this disease every year. Further understanding of the mechanisms that contribute to protective immunity against this parasite will serve to promote the development of an effective vaccine. Here, we describe the importance of the co-stimulatory molecule ICOS during secondary infection with the rodent parasite *Plasmodium chabaudi chabaudi* AS. We show that ICOS promotes the expansion of memory T cells, their acquisition of a secondary follicular helper T (Tfh) cell phenotype, and their ability to provide help to MBCs after reinfection. While ICOS deficient mice control the initial parasite load after re-challenge, the absence of ICOS leads to higher relapsing parasitemia compared to wild-type mice. We establish that the lack of expansion of effector cells with a Tfh cell phenotype in *Icos*^-/-^ mice prevents germinal center formation after secondary infection. Thus, we show that ICOS signaling in T cells promotes an effective memory T cell response and suggests that the enhancement of this co-stimulatory pathway during vaccination may enhance protective immunity to blood-stage *Plasmodium* infection.

## Introduction

Malaria infects over 219 million people each year, with more than 400,000 people reported to have succumbed to the disease in 2017 [1]. While the risk of death declines with the rate of exposure and age, people living in endemic areas suffer symptoms of uncomplicated malaria until immunity against disease develops. Although immune mediators, such as antibodies (Abs) and memory B and T cells, are generated during infection and maintained for months between individual infections, sterile immunity is never achieved. Understanding the factors in the host and parasite that contribute to the inefficient development of immunity is a major hurdle that needs to be overcome to eradicate malaria. Dysregulation of the host immune response through the generation of atypical memory B cells (MBCs) or favoring the activation and expansion of memory T cells that are predisposed to take on a Th1 phenotype over those that generate follicular helper (Tfh) cells, which are capable of supporting Ab production, are possible mechanisms that contribute to the inefficient acquisition of *Plasmodium*-specific immunity [2–6].

Memory T cells are antigen-experienced T cells that survive the contraction phase of a primary immune response. These T cells are responsible for the rapid activation of the adaptive immune response upon secondary encounter with antigen. Populations of CD4^+^ memory T cells are distinguished from one another based on localization and phenotype and include effector (T_EM_; CD44^hi^CD62L^lo^CCR7^-^), central (T_CM_; CD44^hi^CD62L^hi^CCR7^+^IL-7Rα^hi^), and tissue-resident (T_RM_; CD62L^lo^IL-7Rα^int/-^CD69^+^CD122^lo^) memory [7,8]. These memory T cells largely re-assume their original effector identity or exhibit considerable plasticity during antigenic restimulation [6,7,9–12]. However, the nature of memory Tfh cells and their place within this paradigm is less understood. Indeed, despite a large body of research indicating that memory Tfh cells can be derived from primary Tfh effectors [10,11,13–20], the precise sequence of events leading to fate determination has yet to be fully defined.

Previous work has implicated ICOS-ICOSL interactions in Tfh cell differentiation after primary immunization or infection [21–25]. However, information regarding whether ICOS expression by memory CD4^+^ T cells has a direct effect on the ability of these cells to adopt a Tfh phenotype in response to antigen re-challenge is limited, specifically in the context of infection. Despite this knowledge gap, defective ICOS signaling was shown to influence the production [10,26,27], maintenance [27–29], and reactivation [30] of memory CD4^+^ T cells, and in one case the formation of secondary GCs [31]. These results suggest that there are multiple points prior to or after antigen re-encounter in which ICOS signaling could influence secondary T cell responses, including Tfh cell expansion.

Here we were interested in determining the role of ICOS in the secondary immune response to blood-stage *P*. *c*. *chabaudi* AS infection, specifically whether its expression is required for memory T cells to adopt a Tfh cell effector phenotype after recall. Using ICOS deficient mice or an Ab to block ICOS-ICOSL interactions in wild-type (WT) mice, we show that ICOS signaling is important for the expansion of T cells with a Tfh-like cell phenotype and GC formation in the secondary response. Results that were confirmed with the adoptive transfer of *Icos*^-/-^ T_CM_ cells into *tcrb*^*-/-*^ mice that were then challenged with *P*. *c*. *chabaudi*. Additionally, the lack of ICOS led to a reduction in antibody-secreting cells (ASCs) shortly after reinfection, indicating that ICOS signaling in T cells is necessary to promote the differentiation of MBCs into ASCs in recall responses. Collectively, our data suggest that ICOS signaling in memory T cells is necessary to promote short and long-term Ab production after reinfection with *P*. *c*. *chabaudi*.

## Results

### Recrudescent parasitemia influences memory T cell numbers in *Icos*^-/-^ mice after *Plasmodium* infection

**Fig 1:**
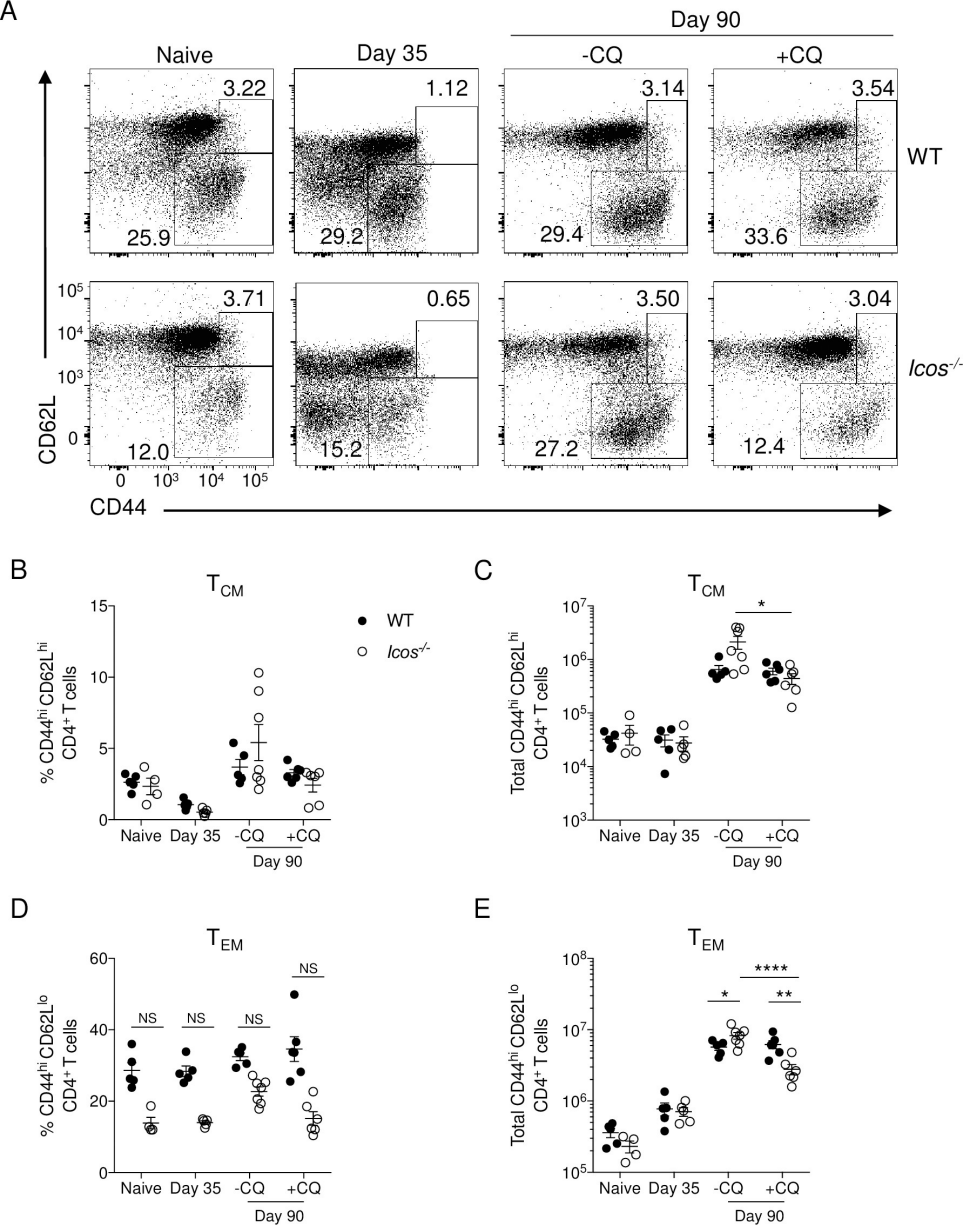
Elimination of persistent *P*. *c*. *chabaudi* infection decreases memory T cell numbers in *Icos*^-/-^ mice. WT and *Icos*^*-/-*^ mice were infected with 10^5^ *P*. *c*. *chabaudi* AS parasitized red blood cells (pRBCs). Wild-type (WT) C57BL/6 or *Icos*^*-/-*^ mice were administered chloroquine (40 mg/kg; +CQ) or 0.9% NaCl (-CQ) i.p. for 10 days beginning on day 35 p.i. Mice were sacrificed on day 35 or 90 p.i. Naïve WT and *Icos*^*-/-*^ mice served as additional controls. (**A**) Representative flow plots of CD44 and CD62L expression on live CD4^+^ T cells from WT and *Icos*^*-/-*^ mice before (day 35) or after treatment with (+CQ) or without (-CQ) chloroquine on day 90 p.i. Frequency (**B**) and total number (**C**) of T_CM_ (CD44^hi^CD62L^hi^) cells as defined by the upper gated box in panel A. Frequency (**D**) and total number (**E**) of T_EM_ (CD44^hi^ CD62L^lo^) cells as defined by the lower gated box in panel A. Data are representative of three independent experiments with 4–6 mice per group (error bars, s.e.m.). An aligned rank transformation was performed on non-parametric data before determining significance by two-way ANOVA with a post hoc Holm-Sidak’s multiple comparisons test. * *p* < 0.05, ** *p* < 0.01, **** *p* < 0.0001, NS not significant.

To determine if the absence of ICOS affects the ability of mice to generate memory CD4^+^ T cells after *P*. *c*. *chabaudi* infection, WT, and *Icos*^-/-^ mice were examined for the presence of T_CM_ and T_EM_ cells based on a CD44^hi^CD62L^hi^ or CD44^hi^CD62L^lo^ phenotype, respectively. *P*. *c*. *chabaudi* AS infection induces an acute parasitemia that is typically resolved within three to four weeks post-infection (p.i.). However, a recrudescent, low burden infection persists in WT C57BL/6 mice for several months, only resolving fully between 90–100 days p.i. Following control of the acute infection, a relatively small frequency of T_CM_ cells were present in the spleen of WT and *Icos*^-/-^ mice at day 35 p.i. and their numbers were comparable to that seen in naïve mice (**Fig 1A–1C**). Although there was a higher frequency of T_EM_ cells in the spleen of WT mice at day 35, no difference in cell numbers was seen at this time (**Fig 1A and 1D and 1E**). By day 90 p.i. a discernable and comparable population of T_CM_ cells was present in the spleen of WT and *Icos*^-/-^ mice, but there was a lower frequency of T_EM_ cells in the *Icos*^-/-^ mice (**Fig 1A, 1B and 1D**). While no significant difference in T_CM_ cell numbers was observed at day 90, T_EM_ cells were significantly increased in the *Icos*^-/-^ mice compared to WT mice at this time (**Fig 1C and 1E**).

As *Icos*^-/-^ mice, but not WT mice, continue to display a relapsing parasitemia at day 90 p.i. [32] it was of interest to establish if the persistent infection influenced the ability of *Icos*^-/-^ mice to maintain similar memory T cell counts at this time. Therefore, WT and *Icos*^-/-^ mice were treated with chloroquine (CQ) to eliminate recrudescent parasites starting on day 35 p.i. and were subsequently evaluated on day 90 p.i.– 45 days after the last CQ dose was administered. CQ treatment caused no change in the percentage or number of T_CM_ cells in the spleen of WT mice at day 90, but it did significantly reduce the number, but not frequency, of T_CM_ cells in the spleen of *Icos*^-/-^ mice (**Fig 1A–1C**). Furthermore, the elimination of parasites with CQ reduced the frequency and significantly reduced the total number of T_EM_ cells in *Icos*^*-/-*^ but not WT mice at day 90 (**Fig 1A and 1D–1E**).

As an alternative to using CD44 and CD62L to define CD4^+^ T cells responding to *P*. *c*. *chabaudi* infection we used the upregulation of two surrogate-activation markers: CD11a and CD49d to examine differences in antigen-experienced CD4^+^ T cells between WT and *Icos*^-/-^ mice. This approach has been confirmed for viral infection [33] and other models of rodent malaria [34–36]. In the absence of CQ, there was a similar percentage and number of CD49d^+^CD11a^+^ CD4^+^ T cells in the spleen of WT and *Icos*^-/-^ mice (**S1 Fig**). In the presence of CQ, there was a significant increase in the proportion of CD49d^+^CD11a^+^ CD4^+^ T cells in WT but not *Icos*^-/-^ mice at day 90. However, CQ treatment caused a significant decrease in the number of CD49d^+^CD11a^+^ CD4^+^ T cells in WT and *Icos*^-/-^ mice with a greater reduction occurring in *Icos*^-/-^ mice. Examination of the CD49d^+^CD11a^+^ CD4^+^ T cells for CD44 and CD62L expression indicated that the loss in cell numbers with CQ treatment was reflected primarily in the T_EM_ cells, as a significant decrease in T_EM_ cells occurred in both groups with a larger reduction seen in ICOS deficient mice. Hence, the total number of CD49d^+^CD11a^+^ CD4^+^ T cells and T_EM_ cells within this antigen-experienced population were significantly lower in *Icos*^-/-^ mice compared to WT mice with the administration of chloroquine.

Together these data indicate that CD4^+^ T cells with an effector phenotype are maintained at an elevated rate, particularly in the absence of ICOS in response to persistent infection with *P*. *c*. *chabaudi*. Overall, the data indicate that mice can generate and maintain populations of T_CM_ and T_EM_ cells in the spleen in the absence of ICOS after *P*. *c*. *chabaudi* infection. While we cannot rule out that CQ treatment negatively impacts memory T cell formation, the elimination of an antigen source, in this case, influenced the ability of mice, especially in the absence of ICOS, to generate memory T cell populations after infection. Therefore, based on the above findings, additional evaluation of the memory response in these mice after drug treatment is warranted.

### Tfh cells fail to expand in *Icos*^-/-^ mice after secondary *P*. *c*. *chabaudi* infection

**Fig 2:**
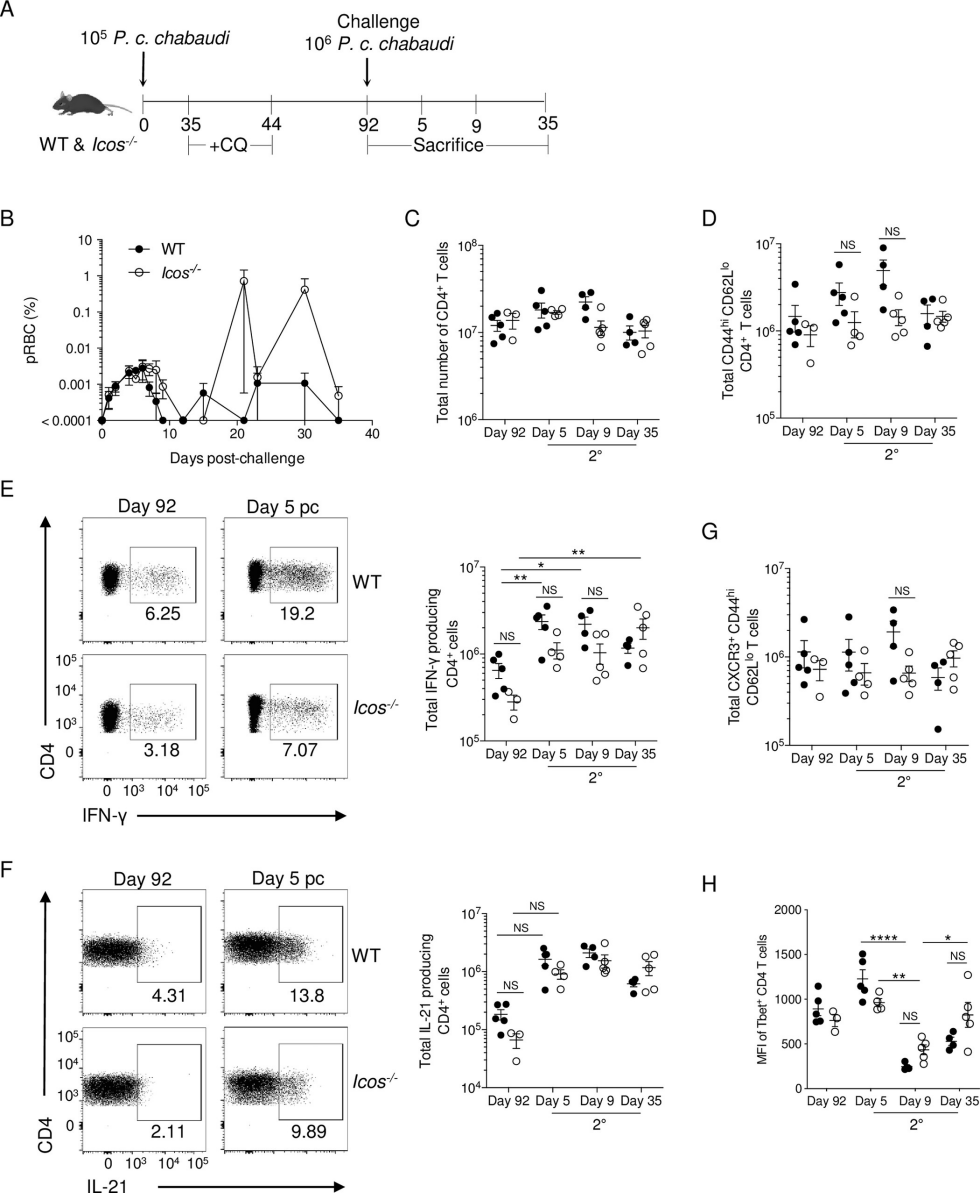
The absence of ICOS does not modulate the Th1 response after re-challenge. **(A)** Experimental model. WT and *Icos*^*-/-*^ mice were infected with 10^5^ *P*. *c*. *chabaudi* pRBCs and CQ was administered to all mice beginning on day 35 p.i. On day 92, a small cohort of mice was sacrificed. The remaining mice were re-challenged with 10^6^ *P*. *c*. *chabaudi* pRBCs i.p. and sacrificed on days 5, 9, and 35 p.c. **(B)** Representative parasitemia curve following re-challenge of WT and *Icos*^*-/-*^ mice. Total number of live CD4^+^ **(C)** and effector CD4^+^ (CD44^hi^CD62L^lo^) **(D)** T cells per spleen at the indicated time points. Representative plots and total number of IFN-γ^+^ **(E)** and IL-21^+^ **(F)** CD4^+^ T cells after *ex vivo* stimulation with PMA and ionomycin in the presence of Brefeldin A. **(G)** Total number of live CXCR3^+^ effector CD4^+^ T cells. (**H**) Median fluorescent intensity (MFI) of Tbet in effector CD4^+^ T cells. Data are representative of two independent experiments with at least three mice per group (error bars, s.e.m.). An aligned rank transformation was performed on non-parametric data before determining significance by two-way ANOVA with a post hoc Holm-Sidak’s multiple comparisons test. * *p* < 0.05, ** *p* < 0.01, **** *p* < 0.0001, NS not significant.

Whether ICOS signaling promotes the acquisition of a Tfh cell effector phenotype from memory T cells or serves additional roles in the secondary response is unclear. Therefore, to investigate the role of ICOS in a secondary response, drug-cleared WT and *Icos*^-/-^ mice were re-challenged with 10^6^ *P*. *c*. *chabaudi* infected red blood cells, a log higher than the primary infection, on day 92 p.i. and the resulting blood parasitemia was monitored (**Fig 2A and 2B**). The initial parasite load peaked at the same time post-challenge (p.c.) in WT and *Icos*^*-/-*^ mice, but clearance of the acute infection was delayed in *Icos*^*-/-*^ mice. Furthermore, although parasitemia relapsed and remitted in both groups of mice, the relapsing parasitemia was higher in *Icos*^*-/-*^ mice, indicating that they are deficient in their ability to control the persistent stage of infection.

Neither the total CD4^+^ T cells nor the number of effector (CD44^hi^CD62L^lo^) CD4^+^ T cells were significantly different between WT and *Icos*^-/-^ mice after re-challenge (**Fig 2C and 2D**). Although, a greater expansion of effector T cells on average was seen in WT mice on days 5 and 9 after re-challenge. Furthermore, there was a rapid expansion of IFN-γ–producing CD4^+^ T cells in WT and *Icos*^-/-^ mice at day 5 p.c., and the total number of IFN-γ^+^ CD4^+^ T cells was not significantly different between WT and *Icos*^-/-^ mice after re-infection (**Fig 2E**). Overall, IFN-γ–producing CD4^+^ T cells remained present in the spleen of WT and *Icos*^-/-^ mice at day 35 p.c., as the maintenance in IFN-γ production may contribute to the control of the relapsing infection after re-challenge (**Fig 2B and 2E**). Similar to IFN-γ, a rapid expansion in IL-21–secreting CD4^+^ T cells was noted at day 5 p.c. in WT and *Icos*^-/-^ mice, their numbers were comparable and remained elevated throughout the secondary response compared to before re-challenge (**Fig 2F**).

The production of IFN-γ is typical of a Th1 effector response, while the expression of IL-21 is associated with Tfh cells; however, IL-21 expression was also shown to be associated with effector cells displaying a mixed Th1/Tfh cell phenotype after primary infection with *P*. *c*. *chabaudi* [37]. Indeed, effector CD4^+^ T cells co-expressing IFN-γ and IL-21 were also present after re-challenge in WT and *Icos*^-/-^ mice (**S2A Fig**). Moreover, there was no significant difference in the number of IFN-γ^+^IL-21^+^ double producers in WT and *Icos*^-/-^ mice. However, their numbers were lower on average in *Icos*^-/-^ mice prior to re-infection. Examination of other markers associated with Th1 effector cells, including CXCR3, a chemokine receptor involved in the trafficking of Th1 cells, indicated that WT and *Icos*^-/-^ splenic CD4^+^ T cells express this receptor before and after re-challenge with no significant difference in its expression seen between these groups of mice (**Fig 2G and S2B Fig**). Also, the median fluorescent intensity (MFI) of Tbet expression in CD4^+^ T cells was comparable between WT and *Icos*^-/-^ mice before and after re-infection. However, the expression of this transcription factor was significantly downregulated at day 9 p.c. before increasing again at day 35 p.c. in WT and *Icos*^-/-^ mice (**Fig 2H and S2C Fig**).

Furthermore, while the frequency of Foxp3^+^ expressing regulatory T cells (Tregs) was significantly higher in WT mice after re-challenge, total numbers of these cells were comparable between WT and *Icos*^-/-^ mice (**S2D–S2F Fig**), which was similar to our results from the primary infection [32]. However, unlike the primary infection [32], *Icos*^-/-^ mice do not show an enhanced Th1 response after secondary infection with *P*. *c*. *chabaudi*. Instead, the magnitude of the Th1 response resembles that seen in WT mice.

**Fig 3:**
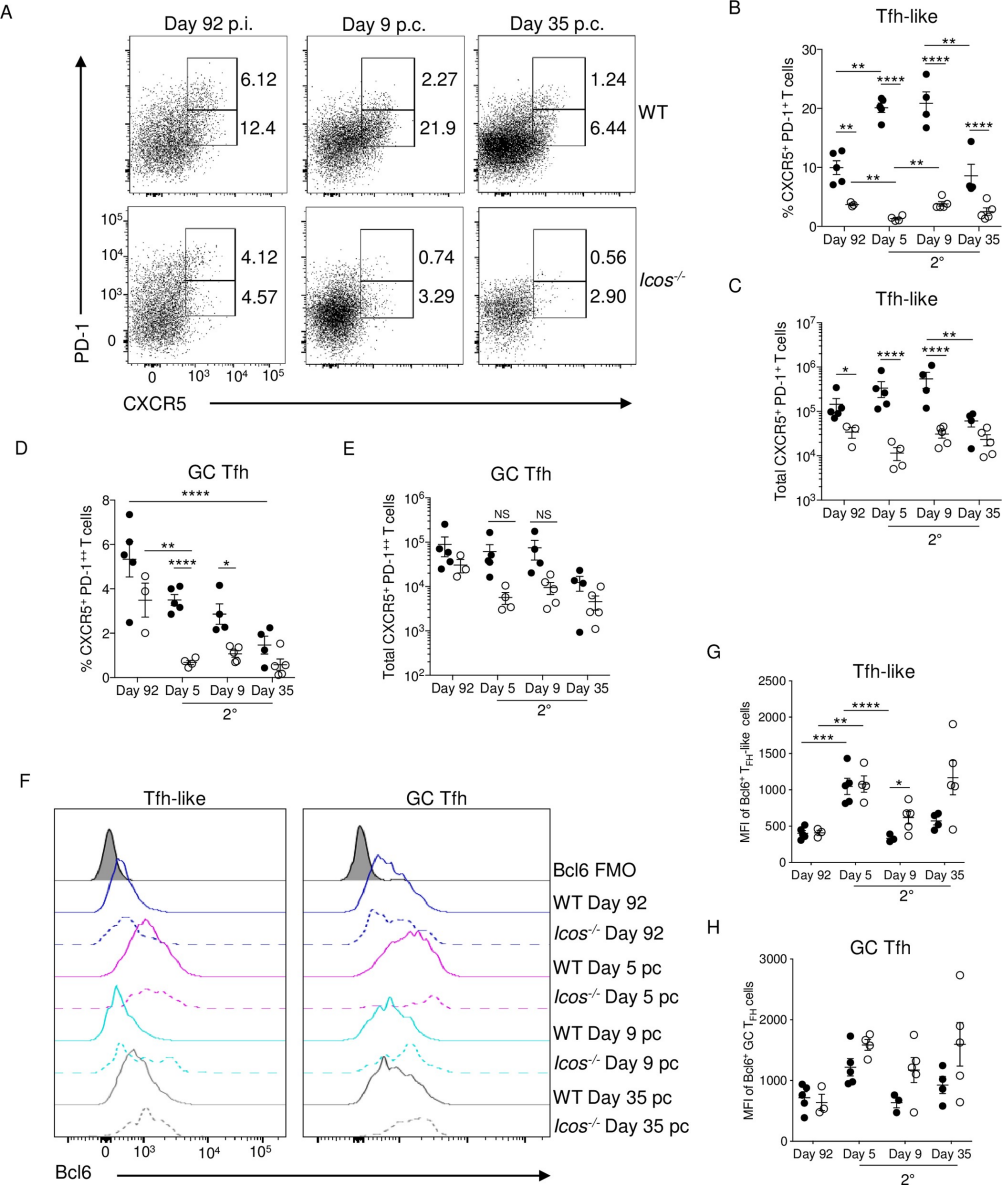
Tfh-like cells fail to expand in *Icos*^*-/-*^ mice after re-challenge. **(A)** Cumulative data showing CXCR5 and PD-1 expression on live, CD44^hi^CD62L^lo^ effector CD4^+^ T cells. The upper box represents GC Tfh (CXCR5^+^PD-1^++^) cells, and the lower box is Tfh-like (CXCR5^+^PD-1^+^) cells. The percentage **(B)** and total number **(C)** of Tfh-like cells in WT and *Icos*^*-/-*^ mice at the indicated times. The percentage **(D)** and total number **(E)** of GC Tfh cells in WT and *Icos*^*-/-*^ mice at the indicated times. **(F)** Histogram of Bcl6 expression in Tfh-like and GC Tfh cells at the indicated timepoints. MFI (median) of Bcl6 expression in **(G)** Tfh-like and **(H)** GC Tfh cells. Data are representative of two independent experiments with at least three mice per group (error bars, s.e.m.). An aligned rank transformation was performed on non-parametric data before determining significance by two-way ANOVA with a post hoc Holm-Sidak’s multiple comparisons test. * *p* < 0.05, ** *p* < 0.01, *** *p* < 0.001, **** *p* ≤ 0.0001, NS not significant.

Tfh cells were evaluated before and after re-challenge of WT and *Icos*^*-/-*^ mice to determine if the absence of ICOS impairs the ability of CD4^+^ T cells to promote a humoral response after re-infection. Two populations of Tfh cells are present in the spleen before re-challenge: CXCR5^+^PD-1^++^ GC Tfh cells that represent fully differentiated Tfh cells and CXCR5^+^PD-1^+^ Tfh-like cells that consist of immature Tfh cells that are not localized within the GC [38] (**Fig 3A**). The frequency and number of Tfh-like cells were significantly lower in *Icos*^-/-^ mice before re-challenge. Although not significant, the frequency and the median number of Tfh cells with a GC phenotype were also reduced in ICOS deficient mice (**Fig 3A–3E**). These results were consistent with our previous findings that indicated that *Icos*^-/-^ mice are unable to maintain effector cells with a Tfh phenotype during the persistent stage of infection [32]. After re-challenge, CXCR5^+^PD-1^+^ Tfh-like cells expanded in proportion and number in WT but not *Icos*^*-/-*^ mice. Conversely, the proportion of WT Tfh cells with a GC phenotype contracted after infection, as did the GC Tfh cell population in *Icos*^*-/-*^ mice. However, WT mice did not exhibit a drop in average GC Tfh cell numbers until day 35 p.c., while the reduction in GC Tfh cell numbers occurred more rapidly in *Icos*^*-/-*^ mice (**Fig 3A–3E**).

Bcl6 expression increased in Tfh-like and GC Tfh cells from WT and *Icos*^-/-^ mice after re-infection (**Fig 3F–3H**). Although Tfh-like cell numbers were significantly lower at all times after re-challenge of *Icos*^-/-^ mice, the MFI of Bcl6 expression was similar or higher in *Icos*^-/-^ mice before and after re-challenge (**Fig 3F and 3G**). Likewise, no significant difference in Bcl6 expression by GC Tfh cells was seen between WT and *Icos*^*-/-*^ mice, with the MFI for Bcl6 being higher in the few GC Tfh cells found in *Icos*^*-/-*^ mice after re-challenge compared to those from WT mice (**Fig 3F and 3H**). Overall, Bcl6 expression was higher in the WT and *Icos*^*-/-*^ CD4^+^ T cells with a GC Tfh cell phenotype than those with a Tfh-like phenotype. As *Icos*^*-/-*^ mice have a defect in producing effector cells with Tfh cell phenotypes after re-challenge, we wanted to determine if regulatory Tfh (Tfr) cells that express Foxp3 were also diminished in the absence of ICOS. No significant differences in the proportion of Tfr cells were apparent in the spleen of WT and *Icos*^*-/-*^ mice before and after re-challenge (**S2D, S2G and S2H Fig**). There was however, a significant increase in Tfr cells in WT mice after re-challenge that was not seen in *Icos*^*-/-*^ mice (**S2H Fig**). This significant difference in Tfr cell numbers between WT and *Icos*^*-/-*^ mice did not last long, as their numbers came down at day 9 p.c. in the WT mice.

Together, these results indicate that ICOS expression is essential for the expansion of T cells with a Tfh cell phenotype in a secondary *P*. *c*. *chabaudi* infection. Although, the few Tfh cells (Tfh-like and GC Tfh) that are present in *Icos*^*-/-*^ mice are capable of expressing the Tfh cell-associated transcription factor Bcl6. Also, it does not appear as though new T cells are added to the GC Tfh pool after re-challenge. Surprisingly, GC Tfh cell numbers contract over time after re-infection. However, whether there is turnover within this compartment after a secondary *P*. *c*. *chabaudi* infection is questionable.

### *Icos* deficiency delays antibody production after re-challenge

**Fig 4:**
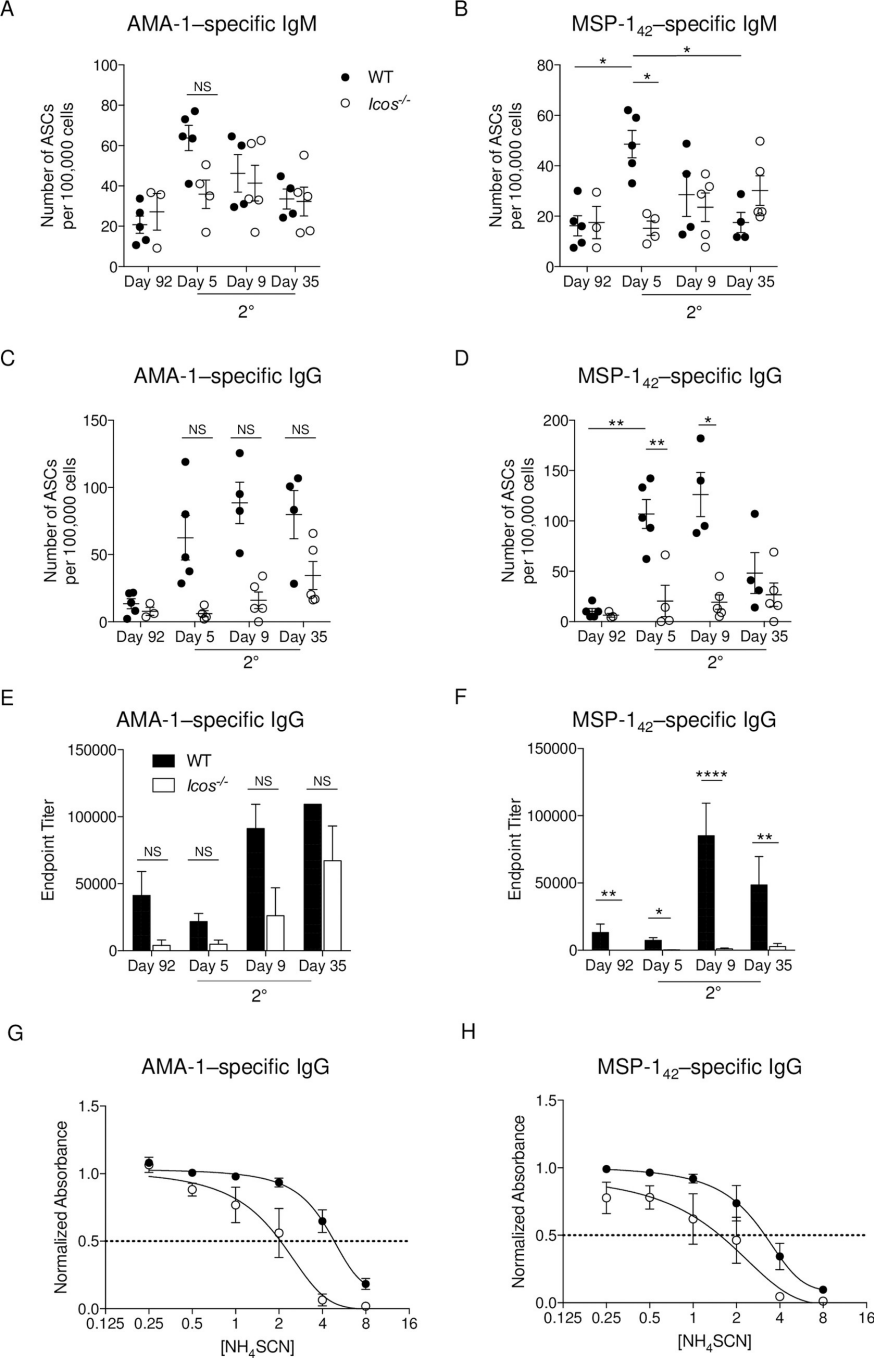
Parasite-specific Ab production is reduced and delayed in *Icos*^*-/-*^ mice after re-challenge. Splenocytes isolated from WT and *Icos*^*-/-*^ mice isolated before re-challenge (day 92) and post-challenge (days 5, 9, and 35) were used to determine the number of parasite-specific IgM **(A, B)** and IgG **(C, D)** ASCs by ELISpot [*P*. *c*. *chabaudi* AMA-1 **(A, C)** and MSP-1_42_ **(B, D)**]. Serum AMA-1 **(E)** and MSP-1_42_ **(F)** specific IgG endpoint titers were determined by ELISA. Normalized absorbance of AMA-1 **(G)** and MSP-1_42_–specific **(H)** IgG at day 35 p.c. as determined by ELISA. The x-axis (log2) displays increasing molar concentrations of NH_4_SCN. A nonlinear regression curve fit is shown; the intersection of the dotted line at the normalized absorbance of 0.5 with the curves represents the concentration of NH_4_SCN needed to elute 50% of the bound IgG off the Ag. Data are representative of two independent experiments with at least three mice per group (error bars, s.e.m.). An aligned rank transformation was performed on non-parametric data before determining significance by two-way ANOVA with a post hoc Holm-Sidak’s multiple comparisons test. * *p* < 0.05, ** *p* < 0.01, **** *p* ≤ 0.0001, NS not significant.

A lack of expansion in Tfh cells in *Icos*^-/-^ mice after secondary infection suggests that Ab production is compromised in the absence of ICOS. Therefore, to evaluate this idea, the humoral response against two common blood-stage antigens–MSP-1 and AMA-1 –was assessed before and after re-challenge in the spleen. Enumeration of IgM-secreting cells specific for AMA-1 and MSP-1 showed an increase in ASCs as early as day 5 p.c. in WT mice (**Fig 4A and 4B**). AMA-1–specific IgM^+^ ASCs also expanded in *Icos*^-/-^ mice at day 5 p.c., but the rate of expansion was lower, and not uniform in every mouse, compared to WT mice. The production of MSP-1–specific IgM^+^ ASCs was delayed in *Icos*^-/-^ mice as their numbers increased in some but not all mice at day 9 p.c. While the number of IgM^+^ ASCs for both antigens was declining by day 35 p.c. in the WT spleens, this population either was maintained or continued to increase in *Icos*^-/-^ mice. An increase in the number of AMA-1 and MSP-1–specific IgG^+^ ASCs, the latter of which was significant, was seen in the spleen at day 5 p.c. in WT but not *Icos*^-/-^ mice (**Fig 4C and 4D**). The number of AMA-1–specific IgG^+^ ASCs increased for some of the *Icos*^-/-^ mice at day 9 p.c., but their median numbers were always lower at all times p.c. compared to WT mice (**Fig 4C**). Meanwhile, only small incremental gains in the number of MSP-1–specific IgG^+^ ASCs were seen in a select few *Icos*^-/-^ mice after re-challenge. The numbers were significantly lower compared to WT mice on days 5 and 9 p.c., and they were only comparable on day 35 p.c. due to a large contraction in numbers in WT mice (**Fig 4D**). Thus, while WT mice rapidly-produce IgM^+^ and IgG^+^ ASCs in the spleen after re-challenge, the production of Ag-specific ASCs is significantly hindered and delayed in *Icos*^-/-^ mice after re-challenge, particularly for class-switched IgG^+^ ASCs.

To further evaluate Ab production, endpoint titers for AMA-1 and MSP-1–specific IgM and IgG were determined using serum from WT and *Icos*^-/-^ mice before and after re-challenge. Before re-challenge, low to negligible titers of AMA-1 and MSP-1–specific IgG were detectable in the serum of *Icos*^-/-^ mice compared to WT mice (**Fig 4E and 4F**). Upon re-challenge, the endpoint titers for AMA-1 and MSP-1–specific IgG decreased on average in WT mice at day 5 p.c. while the IgG titers remained very low to undetectable in *Icos*^-/-^ mice. While AMA-1–specific IgG titers increased in *Icos*^-/-^ mice at day 9 and 35 p.c., they were still lower compared to those seen in WT mice at these times. MSP-1–specific IgG titers increased at day 9 p.c. in WT mice, and they remained elevated at day 35. However, the *Icos*^-/-^ mice continued to display significantly lower endpoint titers for MSP-1–specific IgG at all time points after re-challenge. Moreover, the serum IgG of *Icos*^*-/-*^ mice from day 35 p.c. had a lower affinity for binding its target Ag–AMA-1 or MSP-1 –than its WT counterpart (**Fig 4G and 4H**), suggesting a defect in affinity maturation of the parasite-specific IgG.

Together, these data indicate that a rapid increase in parasite-specific Ab responses occurs locally in the spleen and then systemically in the serum after secondary infection in WT mice. However, the height of Ag-specific Ab production is reduced, and the kinetics of expansion is delayed after re-challenge in the absence of ICOS signaling. Furthermore, affinity maturation of IgG is reduced after secondary infection of *Icos*^*-/-*^mice. Thus, the lack of ICOS signaling negatively impacts the humoral response after secondary *P*. *c*. *chabaudi* infection.

### de novo formation of GCs is impaired in the absence of ICOS after re-challenge

**Fig 5:**
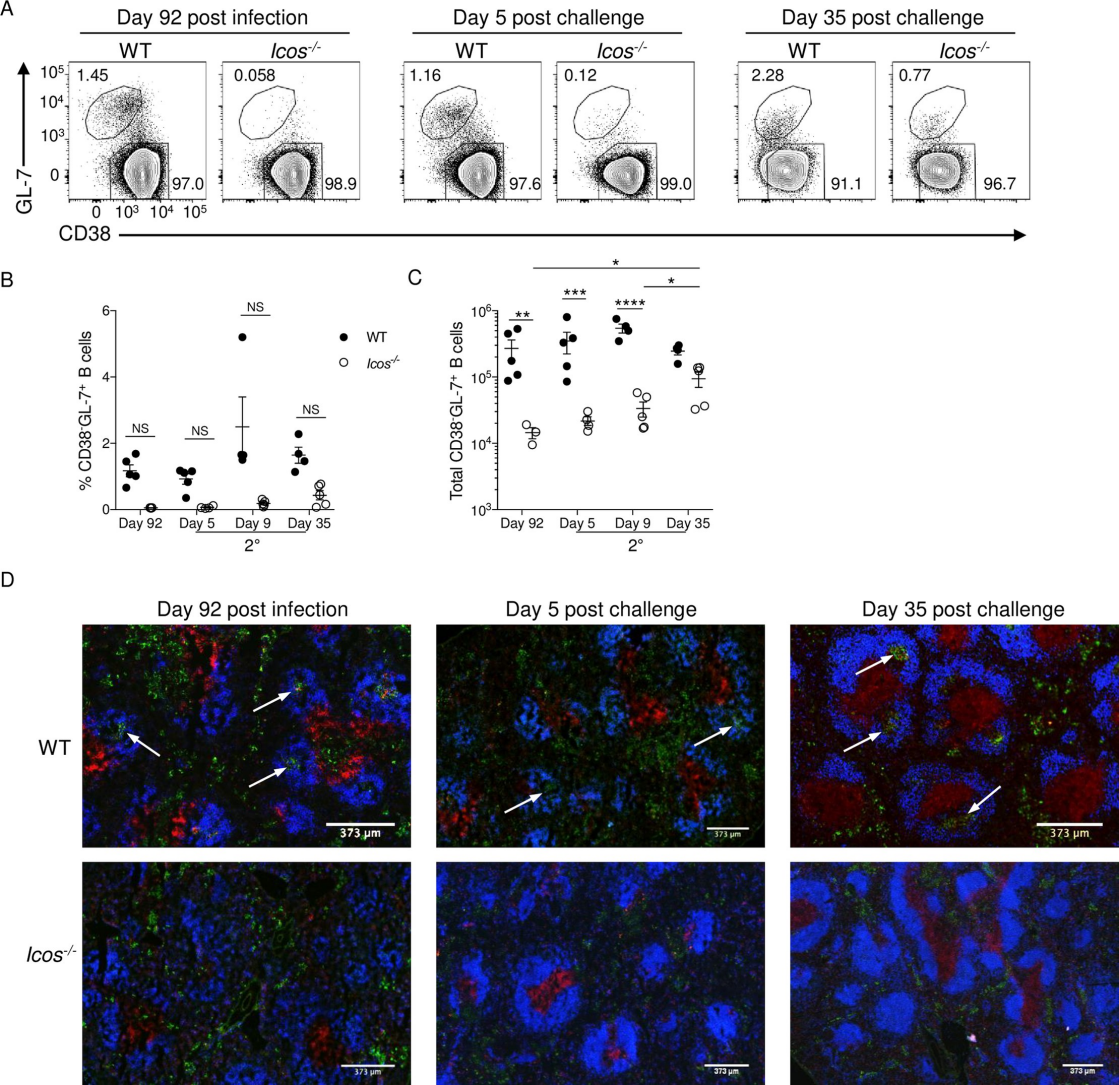
*Icos*^*-/-*^ mice fail to form germinal centers after re-infection. **(A)** Representative plots of CD38 and GL-7 expression on live, CD19^+^B220^+^ B cells from WT and *Icos*^*-/-*^ mice. Upper polygon represents GC B cells (CD38^-^GL-7^+^), and the lower polygon represents naïve/memory B cells (CD38^+^GL-7^-^). **(B)** Frequency and total number **(C)** of GC B cells per spleen at the specified times. **(D)** 4 μm spleen sections from WT and *Icos*^*-/-*^ mice were stained with CD3ε (red), IgD (blue), and GL-7 (green) at the indicated times. White arrows denote representative GCs. Data are representative of two independent experiments with at least four mice per group (error bars, s.e.m.). An aligned rank transformation was performed on non-parametric data before determining significance by two-way ANOVA with post hoc Holm-Sidak’s multiple comparisons test. * *p* < 0.05, ** *p* < 0.01, *** *p* < 0.001, **** *p* ≤ 0.0001, NS not significant.

The observed reduction in GC Tfh cell numbers after re-challenge raises the question as to whether WT and *Icos*^-/-^ mice can mount a GC response after re-infection, or if the secondary response primarily generates just plasmablasts derived from memory and naïve B cells. To detect the presence and kinetics of the GC response, splenocytes from WT or *Icos*^-/-^ mice were examined before and after re-challenge by flow cytometry and immunofluorescence. GC B cells are often identified by flow cytometry as having a CD38^-^GL-7^+^ phenotype. We showed that the absence of ICOS limits the induction of B cells with a GC phenotype after primary *P*. *c*. *chabaudi* infection [32]. Similarly, with CQ treatment, B cells with a GC phenotype were only detectable in the spleen of WT mice at day 92 p.i. (**Fig 5A**). After re-infection, no significant change in the frequency of GC B cells was observed in WT mice (**Fig 5A and 5B**), and there was only a marginal increase in GC B cell numbers after re-challenge (**Fig 5C**). Furthermore, B cells with a GC phenotype were found at a significantly lower abundance in the spleen of *Icos*^-/-^ mice compared to WT mice before and after re-challenge until day 35 p.c. Incremental gains in the number of CD38^+^GL-7^+^ B cells were seen over time in *Icos*^-/-^ mice after secondary infection (**Fig 5A–5C**). However, this late expansion in GC B cells was not uniform across all *Icos*^-/-^ mice, with some mice showing a greater expansion than others.

Visualization of the spleen by immunofluorescence revealed the presence of GC structures within the B cell follicle of WT mice at day 92 p.i. even after CQ treatment. However, as expected, no GCs were seen in the spleen of *Icos*^-/-^ mice at this time, although GL-7^+^ B cells could be seen in the red pulp of both sets of mice (**Fig 5D**). At day 5 p.c., the GC structures remained small in number and size in the spleen of WT mice; instead, the GL-7 staining intensified in the red pulp. The GL-7^+^ cells were only observed in the red pulp of *Icos*^-/-^ mice at this time. Also, some T cell zones became unorganized in the spleen of *Icos*^-/-^ mice with CD3 staining spread throughout the red pulp. By day 35 p.c., GCs became more prominent in WT mice, and the number of GL-7^+^ cells in the red pulp diminished. While there was an increase in GC B cell numbers observed by flow cytometry in *Icos*^-/-^ mice at day 35 p.c. this increase did not correlate with a visual appearance of GC structures within the spleen after re-challenge. The majority of the GL-7^+^ B cells were found outside of the B cell follicle close to CD3^+^ T cells, which were primarily localized in an organized T cell zone, in *Icos*^-/-^ mice (**Fig 5D**). Thus, while GCs are maintained or formed after secondary *P*. *c*. *chabaudi* infection in WT mice, the lack of ICOS prevents the formation of these structures. Instead, it appears that the extrafollicular response in *Icos*^-/-^ mice contributes to the expansion and variation in the humoral response seen after re-infection with *P*. *c*. *chabaudi*. Together these data suggest that defects in the humoral response contribute to the differences in parasitemia seen in *Icos*^-/-^ mice after re-challenge.

### ICOS-ICOSL signaling is crucial for secondary Tfh cell and plasmablast expansion

**Fig 6:**
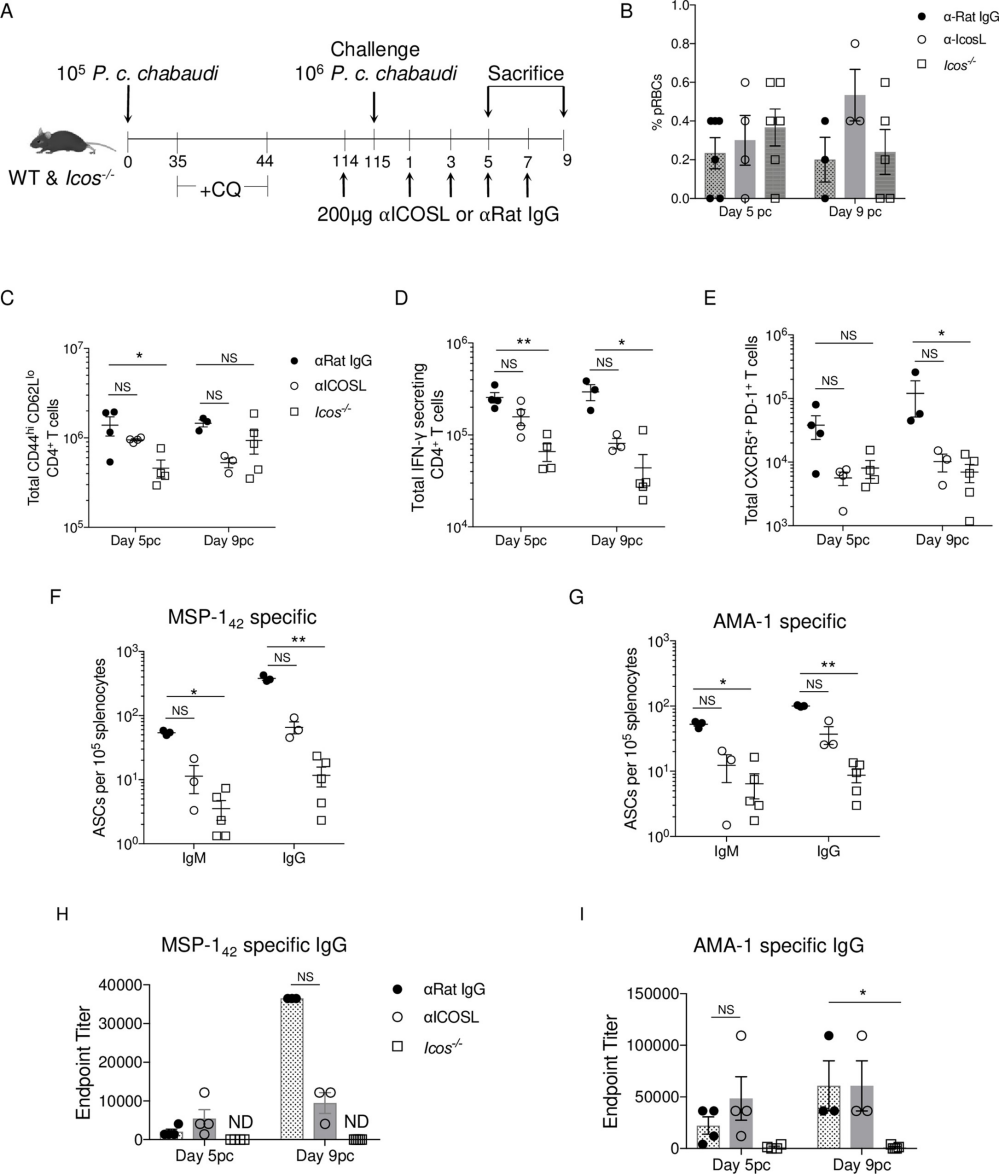
Icos-IcosL signaling promotes the humoral response after re-challenge. **(A)** Experimental model. WT and *Icos*^*-/-*^ mice were infected with 10^5^ *P*. *c*. *chabaudi* pRBCs and given CQ beginning at day 35 p.i. On day 114 p.i. WT mice were treated with 200μg α-IcosL or α-Rat IgG in PBS every other day until day 7 p.c. CQ treated *Icos*^*-/-*^ mice were used as controls. WT and *Icos*^*-/-*^ mice were sacrificed on day 5 or 9 p.c. **(B)** Parasite burden as determined by Giemsa stained thin blood smears on days 5 and 9 p.c. Total number of live CD44^hi^CD62L^lo^ CD4^+^ T cells **(C)**, IFN-γ secreting CD4^+^ T cells **(D)**, and CXCR5^+^PD-1^+^ CD4^+^ cells **(E)** from days 5 and 9 p.c. Number of IgM^+^ and IgG^+^ ASCs per 10^5^ splenocytes specific for *P*. c. *chabaudi* MSP-1_42_ **(F)** and AMA-1 **(G)** on day 9 p.c. Endpoint titers for *P*. *c*. *chabaudi* MSP-1_42_ **(H)** and AMA-1–specific **(I)** IgG on days 5 and 9 p.c. Data are representative of two independent experiments with at least three mice per group (error bars, s.e.m.). Significance calculated by one-way ANOVA Kruskal-Wallis test with post hoc Dunn’s multiple comparisons test. * *p* < 0.05, ** *p* < 0.01, NS not significant.

The early hindrance in Ab production seen in *Icos*^-/-^ mice after re-infection suggests that either the significantly reduced production of class-switched (swIg^+^) MBCs during the primary infection of *Icos*^-/-^ mice (**S3 Fig**), or signaling through ICOS in CD4^+^ T cells after re-infection is contributing to the observed phenotype. To tease apart these two possibilities, WT mice that were treated with CQ were administered either an anti-ICOSL blocking Ab or a rat IgG isotype control every other day starting before and continuing after re-challenge with *P*. *c*. *chabaudi* (**Fig 6A**). Parasite burden in all three groups of mice was comparable on day 5 and 9 p.c. (**Fig 6B**). Blockage of ICOS-ICOSL interactions in WT mice had an intermediate effect on the CD4^+^ T cell and humoral immune response at day 5 and 9 p.c. (**Fig 6C–6I**). For instance, a non-significant reduction in the number of activated (day 5 p.c. *p* = 0.9849; day 9 p.c. *p* = 0.0978), IFN-γ^+^ (day 5 p.c. *p* = 0.2827; day 9 p.c. *p* = 0.2793) and Tfh-like cells (day 5 p.c. *p* = 0.1249; day 9 p.c. *p* = 0.2191) was seen after re-challenge compared to isotype treated mice (**Fig 6C–6E**). Tfh-like cell numbers in the anti-ICOSL treated mice resembled those seen in *Icos*^-/-^ mice.

As a result, fewer antigen-specific IgM^+^ and IgG^+^ ASCs were present in the spleen of the anti-ICOSL treated mice at day 9 p.c. (**Fig 6F and 6G**). The impact of ICOSL blockade was also observed in the serum, as the expansion in MSP-1_42_–specific IgG titers were reduced at day 9 p.c. in the anti-ICOSL treated compared to isotype treated mice (**Fig 6H**). While no difference in AMA-1–specific IgG titers was seen between anti-ICOSL and isotype treated mice at day 9 p.c., a gain in Ab titers for either group was minimal between day 5 and 9 p.c. (**Fig 6I**). Together these data indicate that the defects observed in Ab production in *Icos*^-/-^ mice after re-challenge cannot be solely attributed to a reduction in swIg^+^ MBC production during the primary infection, as blocking ICOS-ICOSL signaling in WT mice during a secondary response diminished Tfh cell and antigen-specific Ab production after re-challenge. Overall, these results indicate that ICOS-ICOSL signaling in CD4^+^ T cells is critical for promoting secondary humoral immune responses to *P*. *c*. *chabaudi*.

### Acquisition of a phenotype indicative of Tfh cells by *Icos*^-/-^ T_CM_ cells is impaired after *P*. *c*. *chabaudi* re-challenge

**Fig 7:**
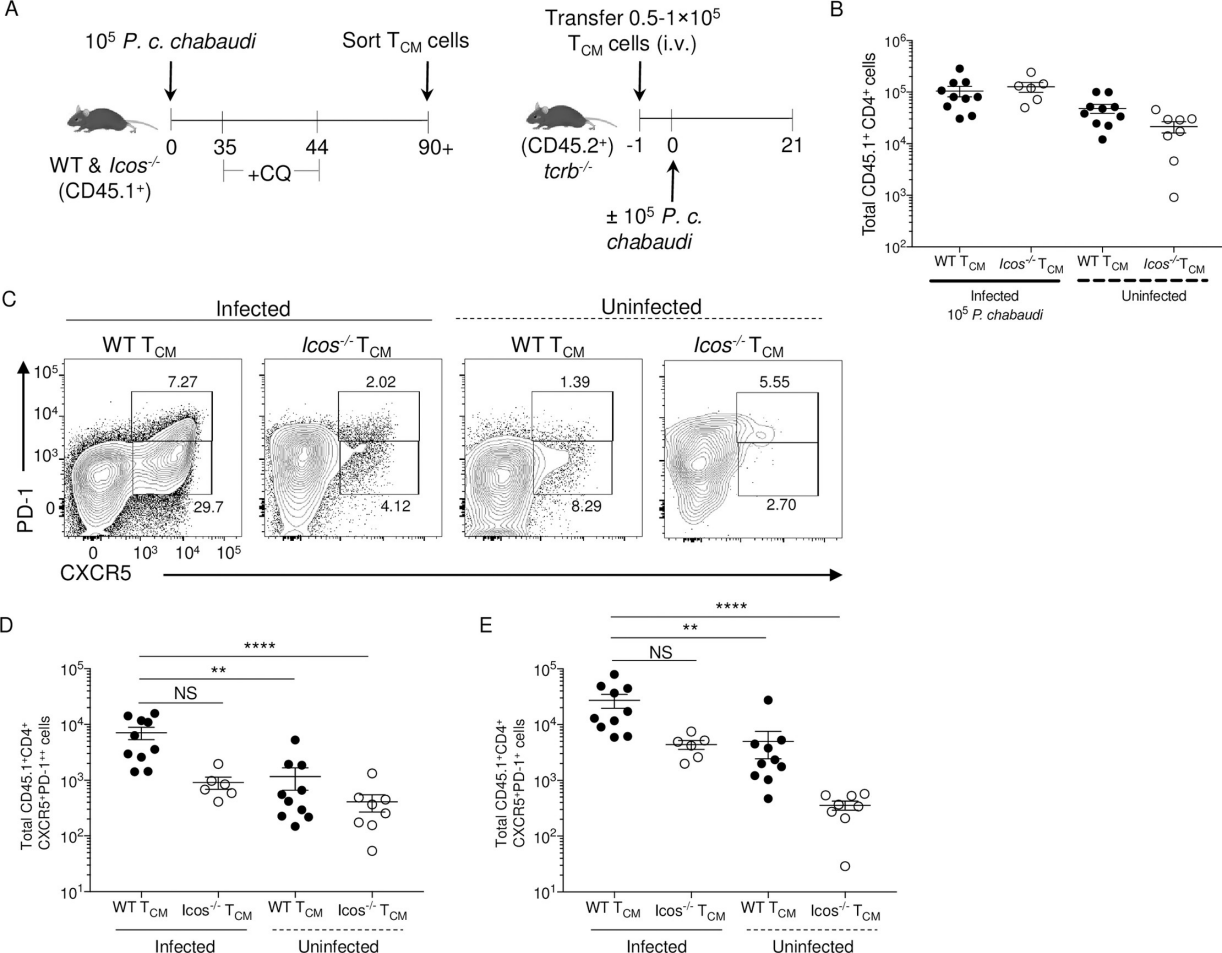
*Icos* deficiency restricts T_CM_ cells from producing Tfh effector cells after reactivation. **(A)** Experimental model. WT and *Icos*^*-/-*^ CD45.1^+^ mice were infected with 10^5^ *P*. *c*. *chabaudi* pRBCs and given CQ beginning at day 35 p.i. T_CM_ cells were sorted from WT and *Icos*^*-/-*^ CD45.1^+^ mice on day 90. 50–100,000 cells of each T_CM_ cell population were transferred retro-orbitally into separate CD45.2^+^ *tcrb*^*-/-*^ mice. WT CD45.2^+^ and *tcrb*^*-/-*^ mice that did not receive donor cells served as controls. Twenty-four hours later, half the mice were infected with 10^5^ *P*. *c*. *chabaudi* pRBCs. Mice were sacrificed at day 21 p.i. **(B)** Total number of live CD45.1^+^ CD4^+^ T cells recovered on day 21 p.i. (day 22 post-transfer). **(C)** Representative plots of CXCR5 and PD-1 expression on live activated (CD44^hi^CD62L^lo^) CD45.1^+^ CD4^+^ T cells at day 21 p.i. Upper gates indicated GC Tfh (CXCR5^+^PD-1^++^) cells, and lower gates represent Tfh-like (CXCR5^+^PD-1^+^) cells. The total number of live activated CD45.1^+^ CD4^+^ **(D)** GC Tfh and **(E)** Tfh-like cells. Data are pooled from two independent experiments with at least three mice per group (error bars, s.e.m.). Significance calculated by one-way ANOVA Kruskal-Wallis test with post hoc Dunn’s multiple comparisons test. ** *p* < 0.01, **** *p* < 0.0001, NS not significant.

To determine if ICOS expression is directly required for T_CM_ cells to take on a Tfh cell phenotype and support humoral immunity, T_CM_ cells were isolated from drug-cleared WT and *Icos*^-/-^ CD45.1^+^ mice at day 90 after *P*. *c*. *chabaudi* infection. Sort-purified T_CM_ cells were transferred into *tcrb*^-/-^ recipients, and the mice were challenged 24 hours later (**Fig 7A**). Parasitemia was monitored in infected mice for 21 days (**S4A Fig**). Although *tcrb*^-/-^ recipients of *Icos*^-/-^ T_CM_ cells had the highest average peak parasitemia at day 9, parasitemia in these mice declined over the next ten days. In contrast, the parasite burden continued to increase from day 9 to 21 in the control *tcrb*^-/-^ mice that received no donor cells. However, by day 21, the parasitemia began to increase again not only in the *tcrb*^-/-^ mice that received *Icos*^-/-^ T_CM_ cells but also those with WT T_CM_ cells, suggesting that these mice will have difficulty controlling the persistent stage of the infection. Experiments were ceased on day 21 p.i. due to the deteriorating health of the control *tcrb*^-/-^ mice.

To control for the homeostatic proliferation that occurs upon adoptive transfer of T cells into a T cell deficit host, *tcrb*^-/-^ mice that received WT or *Icos*^-/-^ T_CM_ cells without subsequent infection were used as a control. Although the number of WT and *Icos*^-/-^ donor CD4^+^ T cells recovered from naïve *tcrb*^-/-^ mice were on average lower than those recovered from infected *tcrb*^-/-^ mice **(Fig 6C**), a higher proportion of them were positive for Ki-67, a marker indicative of cell proliferation (**S4B and S4C Fig**). Further examination of the recovered donor cells indicated that a higher number of the T cells from the infected *tcrb*^-/-^ mice displayed an activated phenotype (CD44^hi^CD62L^lo^) compared to those isolated from the naïve recipients (**S4D Fig**). Overall, there was a comparable number of Ki-67^+^ CD4^+^ T cells displaying an activated phenotype between all groups of mice (**S4E Fig**).

Though the donor T cells displayed an activated phenotype in the spleen of naïve mice, they were mostly unable to co-express CXCR5 and PD-1 (**Fig 7C**). This was not the case after infection as a prominent population of donor WT CD4^+^ T cells co-expressed CXCR5 and PD-1, suggesting a possible Tfh cell fate. However, only a small proportion of *Icos*^-/-^ T cells recovered from infected mice co-expressed CXCR5 and PD-1. Subgating the CXCR5^+^PD-1^+^ T cells into GC (CXCR5^+^PD-1^++^) and Tfh-like (CXCR5^+^PD-1^+^) populations indicated that donor T_CM_ cells from WT and *Icos*^-/-^ mice recovered from naïve *tcrb*^-/-^ mice produced significantly fewer GC Tfh and Tfh-like cells compared to infected *tcrb*^-/-^ mice that received donor WT T_CM_ cells (**Fig 7D and 7E**). Furthermore, there was a reduction, albeit not significant (**Fig 7D** *p* = 0.0942; **Fig 7E** *p* = 0.1713), in the number of GC and Tfh-like cells recovered from the spleen of *tcrb*^-/-^ mice that received ICOS deficient T_CM_ cells compared to those that received WT T_CM_ cells.

Further analysis of the donor cells recovered from the spleen of infected *tcrb*^-/-^ mice indicated that a similar proportion and number of the WT and *Icos*^-/-^ donor T cells produced IFN-γ and IL-21 in response to infection (**S5 Fig**). WT and *Icos*^-/-^ T_CM_ cells generated a comparable number of CXCR5^-^PD-1^+^ effector T cells that were largely CXCR3^+^Tbet^+^, and they were the population that contained the highest frequency and amount of Ly6C^+^ T cells (**S6 Fig**). These are all markers indicative of a Th1 phenotype. Also, the majority of the WT and *Icos*^-/-^ GC Tfh and Tfh-like cells co-expressed CXCR3 and Tbet, but only a small proportion of them also expressed Ly6C. In this case, the number of recovered GC Tfh cells and CXCR3^+^Tbet^+^ GC Tfh cells of ICOS deficient donor status was significantly lower than the cells of WT donor origin. Moreover, a similar pattern in T cell recovery and fate was observed at day 21 p.i. regardless if memory B cells were co-transferred with WT and *Icos*^-/-^ T_CM_ cells (**S7A–S7G Fig**). Also, even though significantly fewer ICOS deficient T cells with a Tfh cell phenotype were recovered from infected *tcrb*^-/-^ mice, similar to our endogenous re-challenge findings, these T cells were equally capable of expressing the Tfh lineage associated transcription factor Bcl6 as those Tfh cells of WT donor origin. (**S7H and S7I Fig**).

Together these data indicate that T_CM_ cells give rise to effector T cells with a Th1 or Tfh cell phenotype upon reactivation, regardless if MBCs are present and that the absence of ICOS limits the generation of effector T cells with a Tfh cell phenotype. Moreover, in this T-cell deficient environment, T_CM_ cells give rise to effector T cells that co-express markers (CXCR5^+^PD-1^+^CXCR3^+^Tbet^+^Bcl6^+^) linked to Th1 and Tfh cells.

### ICOS deficient T_CM_ cells promote a diminished humoral response after *P*. *c*. *chabaudi* challenge

**Fig 8:**
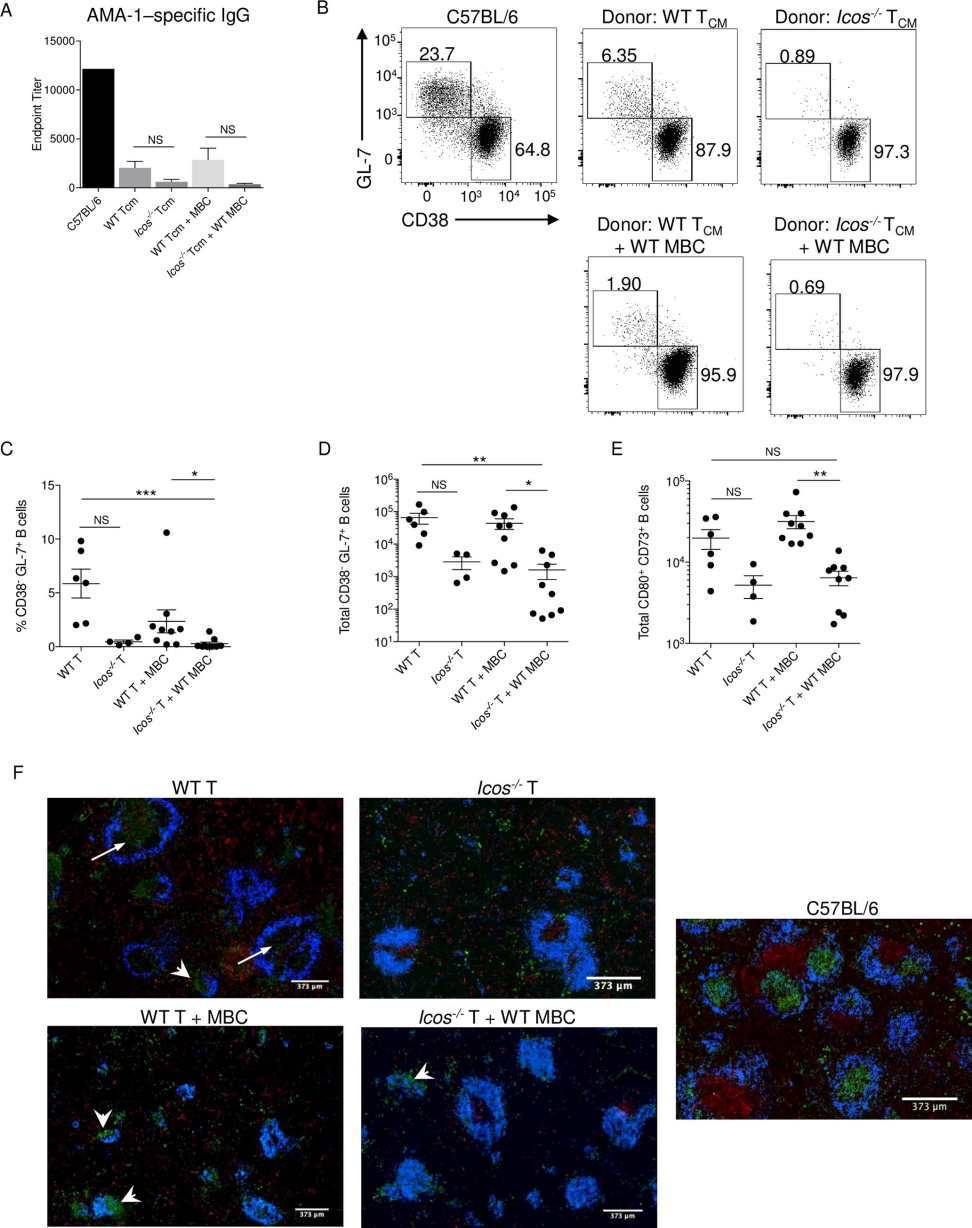
ICOS deficient T_CM_ cells show a defect in B cell help after reactivation. **(A)** AMA-1–specific IgG endpoint titers were determined by ELISA using serum from day 21 p.i. **(B)** Representative dot plots of CD38 and GL-7 expression on live endogenous CD19^+^B220^+^ B cells from *tcrb*^-/-^ mice. Upper boxes represent GC B cells (CD38^-^GL-7^+^), and the lower boxes represent naïve/memory B cells (CD38^+^GL-7^-^). The frequency **(C)** and total number **(D)** of GC B cells from day 21 p.i. **(E)** Total number of CD80^+^CD73^+^CD38^+^GL-7^-^ MBCs on day 21 p.i. **(F)** 4μm spleen sections from day 21 p.i. were stained with CD3ε (red), IgD (blue), and GL-7 (green). White arrows denote GL-7^+^ staining within B cell follicles, and white arrowheads denote clusters of GL-7^+^ cells around B cell follicles. **(A-B, F)** Data are representative of three independent experiments. **(C-E)** Data are pooled from three independent experiments (error bars, s.e.m.). Significance calculated by one-way ANOVA Kruskal-Wallis test with post hoc Dunn’s multiple comparisons test. * *p* < 0.05, ** *p* < 0.01 *** *p* < 0.001, NS not significant.

Since the generation of ICOS deficient T effector cells with a Tfh cell phenotype was limited after transfer and infection, we anticipated that the corresponding humoral response in the *tcrb*^-/-^ recipient mice would be diminished. Indeed, examination of endpoint titers indicated that mice receiving *Icos*^*-/-*^ T_CM_ cells, with or without MBCs, had lower AMA-1–specific IgG titers in their serum than mice receiving WT T_CM_ cells (**Fig 8A**). No MSP-1–specific IgG was detectable in the serum of *tcrb*^-/-^ mice regardless of the donor cells transferred. Furthermore, the emergence of endogenous B cells displaying a GC phenotype (CD38^-^GL7^+^) in *tcrb*^-/-^ mice receiving donor *Icos*^-/-^ T_CM_ cells was diminished after infection regardless if WT MBCs were co-transferred (**S8 Fig**; **Fig 8B–8D**). While *tcrb*^-/-^ mice that received WT T_CM_ cells with or without MBCs produced a similar number of B cells with a GC phenotype. Also, fewer MBCs were produced in *tcrb*^*-/-*^ mice receiving *Icos*^-/-^ T_CM_ cells than mice that received WT T_CM_ cells (**Fig 8E**).

Lastly, analysis of the spleen by immunofluorescence showed that CD3 staining occurred throughout the red pulp and outside of an organized T cell zone in *tcrb*^*-/-*^ mice that received WT T_CM_ cells (**Fig 8F**). A similar pattern of CD3 staining was observed in the red pulp of *tcrb*^*-/-*^ mice that received *Icos*^-/-^ T_CM_ cells. Additionally, loosely organized T cell clusters were present next to B cell follicles, which were often reduced in size, in these inflamed spleens. Compared to intact WT C57BL/6 mice, no well-defined GC structures were visible in the spleen of any recipient *tcrb*^*-/-*^ mice; instead, GL7^+^ B cells were seen throughout the red pulp or concentrated near B cell follicles (arrowheads; **Fig 8F**). There was GL7^+^ staining within some of the follicles in *tcrb*^*-/-*^ mice that received WT T_CM_ cells that may be indicative of GC formation (arrows; **Fig 8F**).

Overall, these results indicate that ICOS sufficient T_CM_ cells are better suited to support the humoral response by promoting B cell differentiation and Ab production after reactivation. However, it does not appear that WT or *Icos*^-/-^ T_CM_ cells are capable of supporting GC formation on their own in the spleen of T-cell deficient mice.

## Discussion

Data presented here demonstrate that the co-stimulatory molecule ICOS is not essential for the generation and maintenance of memory T cells after *P*. *c*. *chabaudi* AS infection. However, upon CQ treatment to remove the persistent primary infection, memory T cell numbers significantly declined in *Icos*^*-/-*^ mice. Re-challenge of intact, drug-cleared WT and *Icos*^*-/-*^ mice led to an impaired expansion of Tfh-like cells in *Icos*^*-/-*^ mice and a reduction in parasite-specific ASCs after re-infection. Consequentially, a corresponding decrease in parasite-specific Ab titers was observed in the serum of *Icos*^*-/-*^ mice. One factor contributing to this defect in Ab production was attributed to a requirement for ICOS-ICOSL signaling after reactivation of memory T cells based on the findings from blocking ICOS-ICOSL signaling in WT mice. Furthermore, GCs were small and loosely organized in the spleen of WT mice prior to and after re-infection, although they became larger and more organized by day 35 p.c. In contrast, GCs were not present in the spleen before or after re-challenge of *Icos*^*-/-*^ mice, suggesting that Ab production was mediated through the extrafollicular response after re-challenge. In support of a role for ICOS in promoting the development of secondary effectors from memory T cells, a larger proportion of WT T_CM_ cells expressed markers associated with Tfh cells after adoptive transfer and infection of *tcrb*^-/-^ mice with *P*. *c*. *chabaudi* compared to those derived from *Icos*^*-/-*^ mice. Collectively, these results suggest that ICOS expression plays an essential role in the development of memory T cells into effector Tfh cells and promoting secondary humoral responses.

Here, we found a higher number of T_CM_ and T_EM_ cells in the spleen at day 90 p.i. in *Icos*^-/-^ mice after *P*. *c*. *chabaudi* infection, results that contrast with other findings. Specifically, the absence of ICOS contributed to a reduction in the generation of *Listeria*-specific T_CM_ cells [10]. While another study indicated that 2WS1 peptide-specific T_EM_ and T_CM_ cell numbers are reduced in *Icosl*^-/-^ mice after infection with *L*. *monocytogenes* engineered to express a 2W1S fusion protein [26]. Whereas other reports indicated that only T_EM_ cell numbers are significantly reduced in the absence of ICOS under homeostatic conditions [28] or in response to influenza infection [27]. In fact, similar to what was observed here, T_CM_ cell numbers were enhanced in *Icos*^-/-^ mice following influenza infection [27]. Evidence in these studies indicates that ICOS signaling is not required to promote the proliferation of memory T cells, as no difference in homeostatic proliferation was found [27]. Nor is ICOS needed to support the survival of memory T cells [26], suggesting ICOS is not required for the formation of the memory T cell pool. However, there is contradictory evidence suggesting that ICOS regulates the survival of resting memory T cells [28].

Nevertheless, the role of ICOS in the formation of memory CD4^+^ T cells appears to be context-dependent and could be influenced by the length of the infection. Influenza and *Listeria* infections are short-lived acute infections that resolve quickly in mice, while persistent-phase *P*. *c*. *chabaudi* infection can last on average, 90 days. Indeed, when CQ was used to eliminate the persistent *P*. *c*. *chabaudi* infection in our model, memory T cell numbers declined in *Icos*^-/-^ mice to those seen in WT mice. Furthermore, when CD11a and CD49d expression were used as a surrogate for denoting antigen-experienced CD4^+^ T cells, a decrease in WT and *Icos*^-/-^ T_CM_ and T_EM_ cells occurred with CQ treatment. Moreover, the decline in cell numbers was greater in the *Icos*^-/-^ mice. These results indicate that the increased availability of antigen and/or the inflammation that perpetuates with parasite recrudescence in non-drug treated mice could lead to the continual generation of newly differentiated T_CM_ and T_EM_ cells. Thus, explaining the heightened memory T cell numbers in these mice compared to the CQ treated group.

Although Majaran et al. reported no defect in memory T cell formation in the absence of ICOS, they did observe a defect in the recall response, as fewer HA19-Env tetramer^+^ CD4^+^ T cells were seen in the draining lymph nodes and spleen on day 5 after boosting with peptide in CFA [30]. Similarly, fewer tetramer-specific hemagglutinin-positive CD4^+^ T cells were observed in *Icosl*^-/-^ mice after challenge with an HA-expressing strain of VSV [27]. We also saw fewer activated T cells in *Icos*^-/-^ mice early after challenge with *P*. *c*. *chabaudi* compared to WT mice, although this difference was not statistically significant. This data supports the findings in other models and indicates that ICOS signaling in memory CD4^+^ T cells under certain circumstances contributes to the reactivation and expansion of these cells. An idea supported by the high frequency of ICOS expression on memory CD4^+^ T cells [28]. This was not the case in the adoptive transfer experiments where reactivation of the polyclonal T_CM_ cells was not impaired in the absence of ICOS. However, we speculate that the lack of competition for Ag with endogenous T cells in the *tcrb*^-/-^ mice negated any requirement for this co-stimulatory molecule in the reactivation and expansion of these cells.

Despite this finding, one commonality in the endogenous re-challenge and adoptive transfer models was that ICOS signaling was required for the T_CM_ cells to adopt a Tfh cell effector phenotype after reactivation. Whereas CD4^+^ T cells that co-express CXCR5 and PD-1 expanded shortly after re-challenge in the intact WT mice, no corresponding increase was observed in *Icos*^-/-^ mice. Similarly, when antigen-specific CD4^+^ memory T cells were adoptively transferred into *Icosl*^*-/-*^ mice and challenged 28 days later, their ability to differentiate into Tfh cells was impaired [26]. In contrast, the ability of CD4^+^ T cells to adopt a Th1 phenotype after reactivation was not impaired, as CD4^+^ T cells from WT and *Icos*^-/-^ mice showed a similar expression profile for the Th1 markers CXCR3 and Tbet.

The inability of memory T cells to adopt a Tfh cell effector phenotype in the absence of ICOS likely compromised their ability to provide help to B cells, particularly MBCs, after re-infection leading to the observed impairment in parasite-specific Ab production. Whether effector T cells need to adopt a Tfh cell phenotype to promote early plasmablast differentiation is debatable, but what is apparent from these studies is that the absence of Tfh cells prevented GC formation in the secondary response in *Icos*^-/-^ mice. However, similar to the primary response [32], *Icos*^-/-^ mice were capable of producing swIg^+^ parasite-specific Abs, albeit with delayed kinetics and at significantly lower titers compared to WT mice. Thus the reduced parasite-specific swIg^+^ Abs, the poor recall response, and the inability to form new GCs during the secondary response allowed the parasite to recrudesce to higher numbers in the *Icos*^-/-^ mice, leading to increased availability of antigens and an inflammatory environment conducive for the potential activation of naïve B cells. Hence, a constant influx of newly activated B cells most likely leads to the continual production of short-lived plasmablasts through the extrafollicular response to limit parasite growth after re-challenge of the *Icos*^-/-^ mice. While the lack of regulatory Tfh (Tfr) cells in *Icos*^-/-^ mice [39] may contribute to the ability of these mice to sustain an extrafollicular B cell response in contrast to their WT counterparts, no defect in the formation of Tfr cells was noted in *Icos*^-/-^ mice before and after re-challenge with *P*. *c*. *chabaudi*. However, unlike what was observed in WT mice, the Tfr population did not expand in *Icos*^-/-^ mice after re-challenge. However, whether this lack of expansion in Tfr cell numbers provides a downstream advantage to the humoral response in ICOS deficient mice is unclear.

It is important to note that Tfh cells that emerge from T_CM_ cells after reactivation do not immediately take on a GC Tfh cell phenotype in response to the infection. While the expansion of Tfh-like cells that express CXCR5 and PD-1 was evident shortly after re-challenge, their numbers declined sharply by day 35 p.c. in WT mice. Conversely, GC Tfh cell numbers did not expand after re-challenge. They steadily declined throughout the secondary infection, which was a surprising result and may indicate that the GC reaction is regulated even more thoroughly in secondary and additional recall responses, possibly by regulatory Tfh cells [40]. It is also unclear if the Tfh cells associated with the secondary GC structures are the same GC Tfh cells that populated the primary GCs, or if new GC Tfh cells derived from reactivated memory T cells promote the formation of new GCs. While we detected CD4^+^ T cells with high expression of PD-1 and CXCR5, a phenotype associated with GC Tfh cells, after challenge, typical GC structures were never visible in the spleen of *tcrb*^-/-^ mice that received WT donor T_CM_ cells. While GC-like structures were present in these recipient mice, their appearance varied from those observed in intact WT mice at this same time with little to no CD3^+^ staining observed within the follicle. One explanation for the atypical architecture found in these mice is that the T-cell deficient environment of the spleen in *tcrb*^-/-^ mice negatively impacted the ability of these mice to form typical GC structures. For starters, the lack of endogenous T cells and the limited amount of memory T cells that were transferred into recipient mice may have put an enormous strain on the reactivated memory T cells to control the infection, causing them to become exhausted before allowing them to differentiate into GC Tfh cells. Another possibility is that the mixed Th1/Tfh-like phenotype favored by the reactivated memory T cells in our model could limit their ability to promote de novo GC formation. Regardless, the origin of the GC Tfh cells that seed the new GCs after a secondary infection is still unclear and requires further investigation.

Lastly, our adoptive transfer studies shed new light on the requirement of MBCs for the reactivation of memory T cells. It was previously reported that MBCs, particularly antigen-specific MBCs, and not CD11c^+^ DCs, are critical for the activation of CXCR5^+^ memory T cells and their acquisition of a Tfh cell effector phenotype [41]. Here we found that bulk T_CM_ cells produced a comparable number of Tfh-like cells in recipient *tcrb*^-/-^ mice when transferred alone or with MBCs. These results suggest that Tfh cells can be derived from memory T cells in the absence of MBCs and that other APCs such as DCs or recently activated naïve B cells can serve to promote reactivation of memory T cells. These results are supported by other findings in which CXCR5^+^ memory T cells were able to recall a Tfh-like response to LCMV in B-cell–deficient mice [11], although this response was diminished compared to WT mice.

The results shown here and by others [26,27,30,31,42] indicate that ICOS signaling is critical for promoting the adoption of a Tfh cell effector phenotype after reactivation of memory T cells, similar to its role in Tfh cell priming during the primary response. Further understanding of how ICOS promotes secondary GC reactions and supports early Ab production in recall responses, specifically the signaling pathways and changes in gene expression regulated by ICOS, will provide us with crucial information for the development of efficacious vaccines.

## Materials and methods

### Ethics statement

All animal studies were done in concordance with the principles set forth by the Animal Welfare Act and the National Institutes of Health guidelines for the care and use of animals in biomedical research. All animal studies were reviewed and approved by the University of Arkansas for Medical Sciences Institutional Animal Care and Use Committee (AUP 3742).

### Mice and infections

Male and female WT (C57BL/6J), CD45.1^*+*^ (B6.SJL-*Ptprc*^*a*^ *Pepc*^*b*^/BoyJ), *Icos*^-/-^ (B6.129P2-Icos^tm1Mak^/J), and *tcrb*^-/-^ (B6.129P2-*Tcrb*^*tm1Mom*^/J) mice were purchased from The Jackson Laboratory. Balb/c mice were obtained from Charles River. *Icos*^*-/-*^ CD45.1^*+*^ mice were generated in house. All mice were housed and bred in specific-pathogen-free facilities at the University of Arkansas for Medical Sciences in accordance with institutional guidelines. Mice were initially infected between six and ten weeks of age. Studies were initially performed utilizing female mice and were replicated using male mice to ensure that the observed results were not a product of a difference in sex. For infection with *Plasmodium chabaudi chabaudi* AS (MRA-741, BEI Resources Repository), male BALB/c mice were infected with parasitized red blood cells (pRBCs) from frozen parasite stocks. Subsequently, 10^5^ pRBCs from each passage were used to intraperitoneally (i.p.) infect experimental mice to establish infection. Challenged mice received 10^6^ pRBCs i.p. Parasitemia during the acute primary infection was determined by flow cytometry [43]. While parasitemia in the persistent stage of the primary infection and after re-challenge was determined by reading Giemsa stained thin blood smears. To eliminate the persistent primary infection, WT and *Icos*^-/-^ mice infected with *P*. *c*. *chabaudi* were treated for ten consecutive days with 40 mg/ kg chloroquine (Sigma) in 0.9% NaCl beginning at day 35 p.i. Mice were subsequently re-challenged at least 45 days after the last dose of chloroquine.

### Flow cytometry and antibodies

To generate a single cell suspension, mouse spleens were passed through a 40 μm filter, and RBCs were lysed in a 0.86% NH_4_Cl solution. Cells were kept in complete RPMI (RPMI 1640, 10% fetal bovine serum, 10% non-essential amino acids, 10% sodium pyruvate, 10% L-glutamine, 10% penicillin and streptomycin, and 1% 2-βME). Cells were washed in FACS buffer (1× PBS, 0.2% BSA and 0.2% 0.5M EDTA), and Fc receptors were blocked with anti-mouse CD16/32 (24G2; BioXCell) in a buffer containing normal mouse and rat IgG (Life Technologies). Abs against CD4, PD-1, Ter119, CD49d, CD11a, B220, CD3ε, CD11b, CD11c, CD44, CD45.1, IFN-γ, TNF-α, CD138, CD80, CD38, GL-7, IgD, IgM, ICOS, SA-APC, SA-BV510, and fixable viability dye were from ThermoFisher or Tonbo Biosciences. Abs against CXCR5, TCR-β, CD19, B220, CD73, and CD44 were from Biolegend, while CXCR5-biotin and CD62L were from BD Biosciences. Following surface staining, samples not requiring intracellular staining were fixed in 4% PFA (Electron Microscopy Sciences). Fluorescence minus one (FMO) controls were used for setting the positive gates and indicating background staining. Samples were run on an LSRIIFortessa (Becton Dickson), and FlowJo 10.3 was used for analysis.

### Intracellular staining

For cytokine staining, splenocytes were incubated with PMA and ionomycin in the presence of Brefeldin A (Sigma) for 4 h at 37°C before surface staining. Cells were fixed with 4% PFA followed by permeabilization using 0.1% saponin diluted in FACS buffer and stained with Abs diluted in this same buffer. For IL-21 identification, cells were incubated with recombinant murine IL-21 receptor (R&D systems) followed by incubation with human Fc receptor coupled to phycoerythrin (ThermoFisher). FMO controls were used for setting the positive gates and indicating background staining.

### Immunofluorescence

Spleens were embedded in OCT, frozen, and cut into 4 μm sections. For staining, each section was fixed in ice-cold 75% methanol/25% acetone for 10 minutes at -20°C. Each slide was blocked with 2% normal goat serum and stained with IgD, CD3ε, and GL-7 (ThermoFisher) overnight at 4°C. Secondary goat anti-rat IgG (H+L)-AF647 and goat anti-Armenian hamster IgG (H+L)-Cy3 were applied for 1h at room temperature followed by an additional stain with a goat anti-rat IgM (μ chain) conjugated to FITC (Jackson Immunoresearch) under the same conditions. Coverslips were mounted using fluoroshield (Electron Microscopy Sciences), and slides were scanned on an EVOS FL Auto2. Images were analyzed using ImageJ (NIH).

### Antibody ELISAs

High binding Immunlon HBX plates (Thermo Scientific) were coated with recombinant *P*. *c*. *chabaudi* AMA-1 or MSP-1_42_ proteins in sodium bicarbonate buffer overnight at 4°C. Plates were blocked with 5% FBS in PBS, and serum was initially diluted 1:50 and then serially diluted 1:3 down the plate. HRP-conjugated IgG (Southern Biotech) was incubated on the plate for 1h at 37°C, and SureBlue substrate (KPL) was used for detection. Plates were read on a FLUOStar Omega plate reader (BMG Labtech) at an absorbance of 450 nm. Endpoint titers were determined as previously described [44]. Briefly, the absorbance (450nm) of five naïve mice at the 1:50 dilution was determined in duplicate for each protein/antibody combo. The standard deviation and degrees of freedom were calculated from the samples. These values, along with the *t* value from *t* distribution table, assuming a 95% confidence interval was inserted into the equation developed by Frey at al. to determine the cut off value [44]. The first dilution at which each sample was below the cutoff is considered the ‘endpoint titer’ for that sample. Each endpoint titer was grouped to graph the mean titer per genotype.

### ELISpot assays

ELISPOT plates (Millipore) were coated with recombinant *P*. *c*. *chabaudi* AMA-1 or MSP-1_42_ protein. 10^5^ splenocytes per well were deposited on the plate and incubated overnight at 37°C. Following washing and blocking, APS conjugated IgM and IgG (Southern Biotech) were applied for 1h at 37°C. Spots were detected using a detection buffer, including NBT and BCIP (Sigma). The plate was read using an AID ELISPOT reader system and analyzed using AID ELISpot 7.0 software (AID GmbH).

### Adoptive transfers

Congenic WT and *Icos*^-/-^ mice were sacrificed at least 1.5 months after drug clearance of *P*. *c*. *chabaudi* parasites. Spleens were harvested and enriched for CD4^+^ T cells and CD73^+^ B cells using an AutoMACS Pro cell separator (Miltenyi). CD4^+^CD44^hi^CD62L^hi^ T_CM_ cells and CD73^+^CD38^+^GL-7^-^ MBCs were sorted using a BD Biosciences FACSAria. The cells were resuspended in PBS, and 0.5–1 × 10^5^ cells of each population were transferred via the retro-orbital sinus into *tcrb*^-/-^ mice. Mice were infected with 10^5^ *P*. *c*. *chabaudi* pRBCs i.p. the following day. Transferred cells were recovered from infected mice on day 21 p.i. utilizing anti-CD45.1-PE, anti-PE microbeads (Miltenyi Biotech), and positive selection on an autoMACs Pro Separator (Miltenyi Biotech).

### α-IcosL blocking

α-IcosL Ab was obtained from BioXCell (BE0028), and α-Rat IgG was obtained from Sigma Millipore. To block ICOS-ICOSL signaling, 200μg of α-IcosL was administered in sterile 1 × PBS every other day to WT mice beginning on the day before re-challenge (day 89 p.i.). α-Rat IgG was administered in the same manner to control WT mice.

### Statistics

GraphPad Prism 7.0 (GraphPad Software, Inc., San Diego, CA) and (R 3.4.3, The R Foundation) were used to perform the statistical analysis. Specific tests of statistical analysis are detailed in the figure legends.

## Supporting information

S1 Fig Chloroquine treatment reduced the number of antigen-experienced CD4^+^ T cells after *P*. *c*. *chabaudi* infection. Representative flow plots of CD49d and CD11a expressing T cells gated through live singlets and non-T lymphocytes were excluded before gating on CD4^+^ T cells **(A)** from naïve or day 90 infected WT and *Icos*^-/-^ mice that were treated with or without CQ. CD49d^+^CD11a^+^ T cells were then further subgated to examine their expression of CD44 and CD62L (**B**). The frequency **(C)** and total number **(D)** of CD11a^+^CD49d^+^ CD4^+^ T cells from naïve and infected WT and *Icos*^-/-^ mice on day 90 p.i. The total number of CD44^hi^CD62L^hi^ CD11a^+^CD49d^+^ T_CM_ cells **(E)** and CD44^hi^CD62L^lo^ CD11a^+^CD49d^+^ T_EM_ cells **(F)** from naïve and infected WT and *Icos*^-/-^ mice on day 90 p.i. Data are representative of two independent experiments with at least three mice per group (error bars, s.e.m.). An aligned rank transformation was performed on non-parametric data before determining significance by two-way ANOVA with a post hoc Holm-Sidak’s multiple comparisons test. * *p* < 0.05, **p < 0.01, **** *p <* 0.0001, NS not significant.
(TIF)

S2 Fig The T_h_1 response and regulatory T cell response are comparable between WT and *Icos*^-/-^ mice. **(A)** Total IFN-γ ^+^IL-21^+^ CD4^+^ T cells after restimulation of splenocytes with PMA and Ionomycin in the presence of Brefeldin A. **(B**) Representative flow plots and frequency of CXCR3-expressing activated CD4^+^ T cells. Splenocytes were gated through live singlets, and non-T lymphocytes were excluded before gating on CD4^+^ T cells. **(C)** Histogram of Tbet^+^ expression by CD4^+^ T cells at the indicated time point. **(D)** Representative flow plots of Foxp3^+^ Tregs and Tfr cells. T cells were gated through live singlets, and non-T lymphocytes were excluded before gating on CD4^+^ T cells. CD4^+^ T cells were then subgated for Foxp3 expression (Tregs) or on CXCR5^+^PD-1^+^ CD4^+^ T cells prior to gating on Foxp3^+^ cells (Tfr). Frequency **(E)** and total number **(F)** of Tregs. Frequency **(G)** and total number **(H)** of Tfr cells. **(A-C)** Data are representative of two experiments with at least three mice per group. **(D-H)** Data are from one experiment with four mice per group (error bars, s.e.m.). An aligned rank transformation was performed on non-parametric data before determining significance by two-way ANOVA with a post hoc Holm-Sidak’s multiple comparisons test. * *p* < 0.05, NS not significant.
(TIF)

S3 Fig Memory B cell numbers are reduced in *Icos*^*-/-*^ mice before re-infection. **(A)** Representative dot plots of CD80 and CD73 expression on live CD38^+^GL-7^-^CD19^+^B220^+^ B cells from WT and *Icos*^*-/-*^ mice derived from the parent gate in Fig 5. Box represents CD80^+^CD73^+^ MBCs. **(B)** Frequency and total number **(C)** of CD80^+^CD73^+^ MBCs per spleen at day 92 p.i. Total number of **(D)** swIg^+^ and **(E)** IgM^+^ CD80^+^CD73^+^ MBCs per spleen at day 92. Data are representative of two independent experiments with at least three mice per group (error bars, s.e.m.). Significance was determined by a Mann-Whitney *t*-test. * *p* < 0.05.
(TIF)

S4 Fig T_CM_ cells expand in *tcrb*^*-/-*^ recipients regardless of infection status. Mice were treated as in Fig 6. **(A)** Parasitemia curve as determined by flow cytometry. **(B)** Representative flow plots of live recovered CD4^+^CD45.1^+^ donor T cells expressing Ki-67. The frequency **(C)** of Ki-67^+^ CD4^+^CD45.1^+^ T cells on day 21 p.i. Total number of activated (CD44^hi^CD62L^lo^) CD45.1^+^CD4^+^ T cells **(D)** and Ki-67^+^ activated T cells **(E)** recovered from *tcrb*^*-/-*^ recipient mice on day 21. Data are pooled from two independent experiments with at least three mice per group (error bars, s.e.m.).
(TIF)

S5 Fig *Icos*^*-/-*^ T_CM_ cells produce IFN-γ and IL-21 after reactivation. **(A)** Representative flow plots of IFN-γ and IL-21-expressing CD45.1^+^CD4^+^ T cells after stimulation with PMA and Ionomycin in the presence of Brefeldin A. Frequency of **(B)** IFN-γ^+^ and IL-21^+^ CD45.1^+^CD4^+^ T cells. Total number of **(C)** IFN-γ^+^, IL-21^+^, and IFN-γ^+^IL-21^+^ CD45.1^+^CD4^+^ T cells. Data are from one experiment with five mice per group (error bars, s.e.m.). Significance was determined by a Mann-Whitney *t*-test.
(TIF)

S6 Fig WT and *Icos*^*-/-*^ T_CM_ cells display a mixed Th1/Tfh-like phenotype after reactivation with *P*. *c*. *chabaudi* infection. WT and *Icos*^*-/-*^ CD45.1^+^CD4^+^ T cells recovered from *tcrb*^*-/-*^ mice on day 21 p.i. were separated into three different gates based on their expression of PD-1 and CXCR5: PD-1^+^CXCR5^-^, Tfh-like (CXCR5^+^PD-1^+^), and GC Tfh (CXCR5^+^PD-1^++^). The three gated populations of T cells were analyzed for Ly6C, CXCR3, and Tbet expression. Graphs represent total numbers of cells for each of the subgated populations of cells. Data are from one experiment with five mice per group (error bars, s.e.m.). Significance was determined by a Mann-Whitney *t*-test. * *p* < 0.05, NS not significant.
(TIF)

S7 Fig *Icos*^*-/-*^ T_CM_ cells fail to adopt a Tfh-like phenotype after co-transfer with MBCs. **(A)** Experimental model. WT and *Icos*^*-/-*^ CD45.1^+^ mice were infected with 10^5^ *P*. *c*. *chabaudi* pRBCs and given CQ beginning at day 35 p.i. T_CM_ cells were sorted from WT and *Icos*^*-/-*^ CD45.1^+^ mice on day 90 along with CD73^+^CD38^+^GL-7^-^ MBCs from WT CD45.1^+^ mice. 100,000 cells of each T_CM_ cell population were transferred together with an equal number of MBCs retro-orbitally into CD45.2^+^ *tcrb*^*-/-*^ mice. WT CD45.2^+^ and *tcrb*^*-/-*^ mice that did not receive donor cells served as controls. Twenty-four hours later, mice were infected with 10^5^ *P*. *c*. *chabaudi* pRBCs. Mice were sacrificed at day 21 p.i. **(B)** Parasitemia curve determined by Giemsa stained thin blood smears. Cross denotes the removal of a morbid mouse from the study. Total number of live **(C)** and activated (CD44^hi^CD62L^lo^) CD45.1^+^CD4^+^ T cells **(D)** recovered from recipient mice on day 21. **(E)** Representative dot plots of CXCR5 and PD-1 expression on live activated CD45.1^+^CD4^+^ T cells at day 21. Polygon identifies the CXCR5^+^PD-1^+^ expressing CD4^+^ T cells. The frequency **(F)** and total number **(G)** of live activated CD45.1^+^CD4^+^ CXCR5^+^PD-1^+^ T cells. Representative histograms and MFI (median) of Bcl6 expression at day 21 p.i. by recovered CXCR5^+^PD-1^+^CD45.1^+^CD4^+^ T cells derived from WT (red peak) or *Icos*^-/-^ (blue peak) donor (H) T_CM_ only and (I) T_CM_ and MBCs groups. Bcl6 FMO (gray peak). Data are pooled from three independent experiments (error bars, s.e.m.). Significance calculated by one-way ANOVA Kruskal-Wallis test with post hoc Dunn’s multiple comparisons test. ** *p* < 0.01, **** *p* ≤ 0.0001.
(TIF)

S8 Fig Gating strategy for endogenous B cells derived from *tcrb*^*-/-*^ mice after transfer of T_CM_ cells and infection. To determine the phenotype of endogenous CD45.2^+^ B cells, splenocytes were gated through live lymphocytes, single cells, dump- (CD3^-^CD11b^-^CD11c^-^Ter119^-^), and subsequently gated on B220^+^CD138^-^ B cells or B220^-^CD138^+^ plasmablasts before reaching the gates displayed in Fig 8.
(TIFF)
