# Supplementary figures and images for: ICOS signaling promotes a secondary humoral response after re-challenge with Plasmodium chabaudi chabaudi AS
Source: PLoS Pathog. 2020 Apr 29;16(4):e1008527. doi: 10.1371/journal.ppat.1008527 (PMC7213745; doi:10.1371/journal.ppat.1008527)

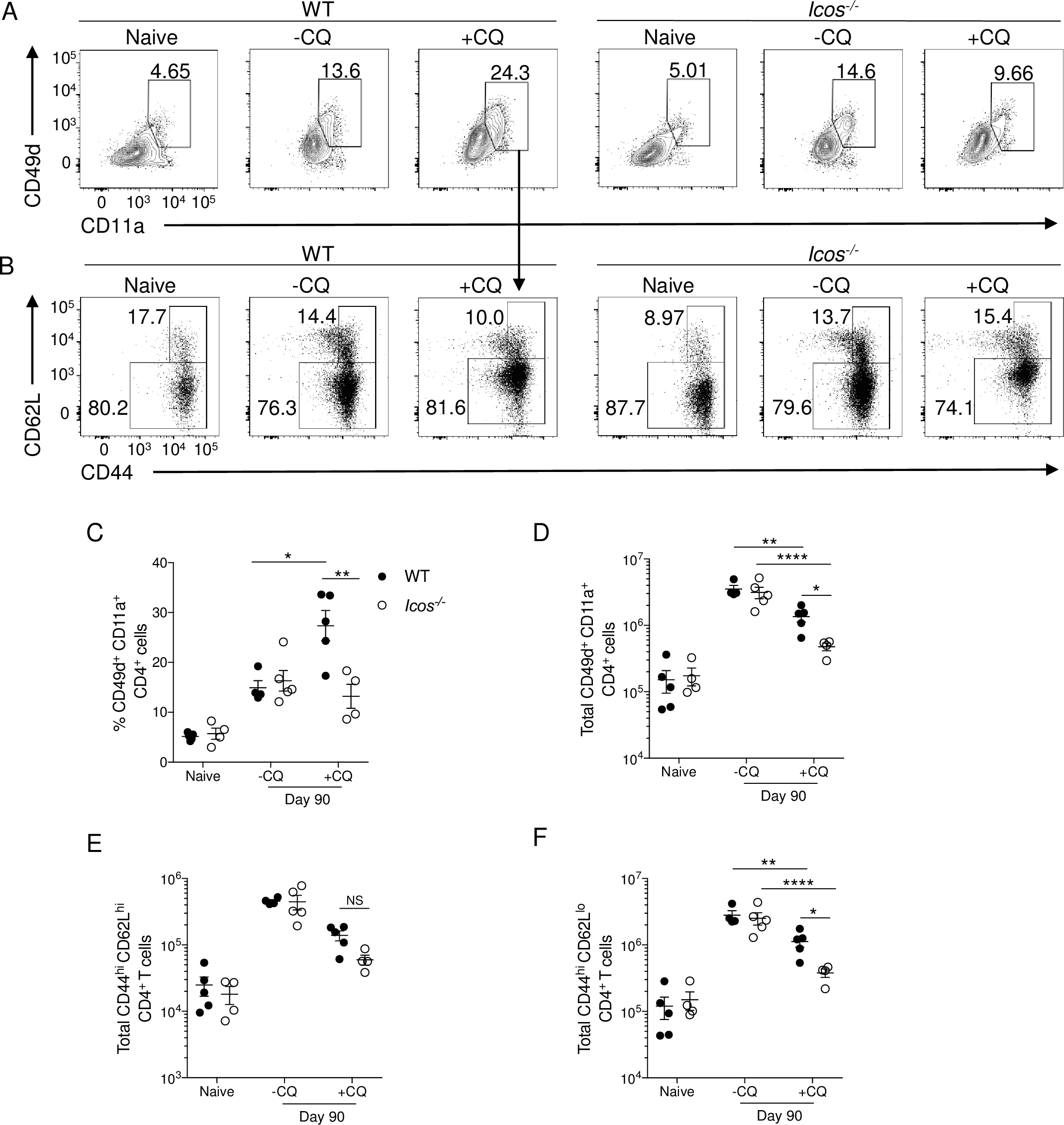

Supplement: S1 Fig — Representative flow plots of CD49d and CD11a expressing T cells gated through live singlets and non-T lymphocytes were excluded before gating on CD4+ T cells (A) from naïve or day 90 infected WT and Icos-/- mice that were treated with or without CQ. CD49d+CD11a+ T cells were then further subgated to examine their expression of CD44 and CD62L (B). The frequency (C) and total number (D) of CD11a+CD49d+ CD4+ T cells from naïve and infected WT and Icos-/- mice on day 90 p.i. The total number of CD44hiCD62Lhi CD11a+CD49d+ TCM cells (E) and CD44hiCD62Llo CD11a+CD49d+ TEM cells (F) from naïve and infected WT and Icos-/- mice on day 90 p.i. Data are representative of two independent experiments with at least three mice per group (error bars, s.e.m.). An aligned rank transformation was performed on non-parametric data before determining significance by two-way ANOVA with a post hoc Holm-Sidak’s multiple comparisons test. * p < 0.05, **p < 0.01, **** p < 0.0001, NS not significant. (TIF) [file ppat.1008527.s001.tif]

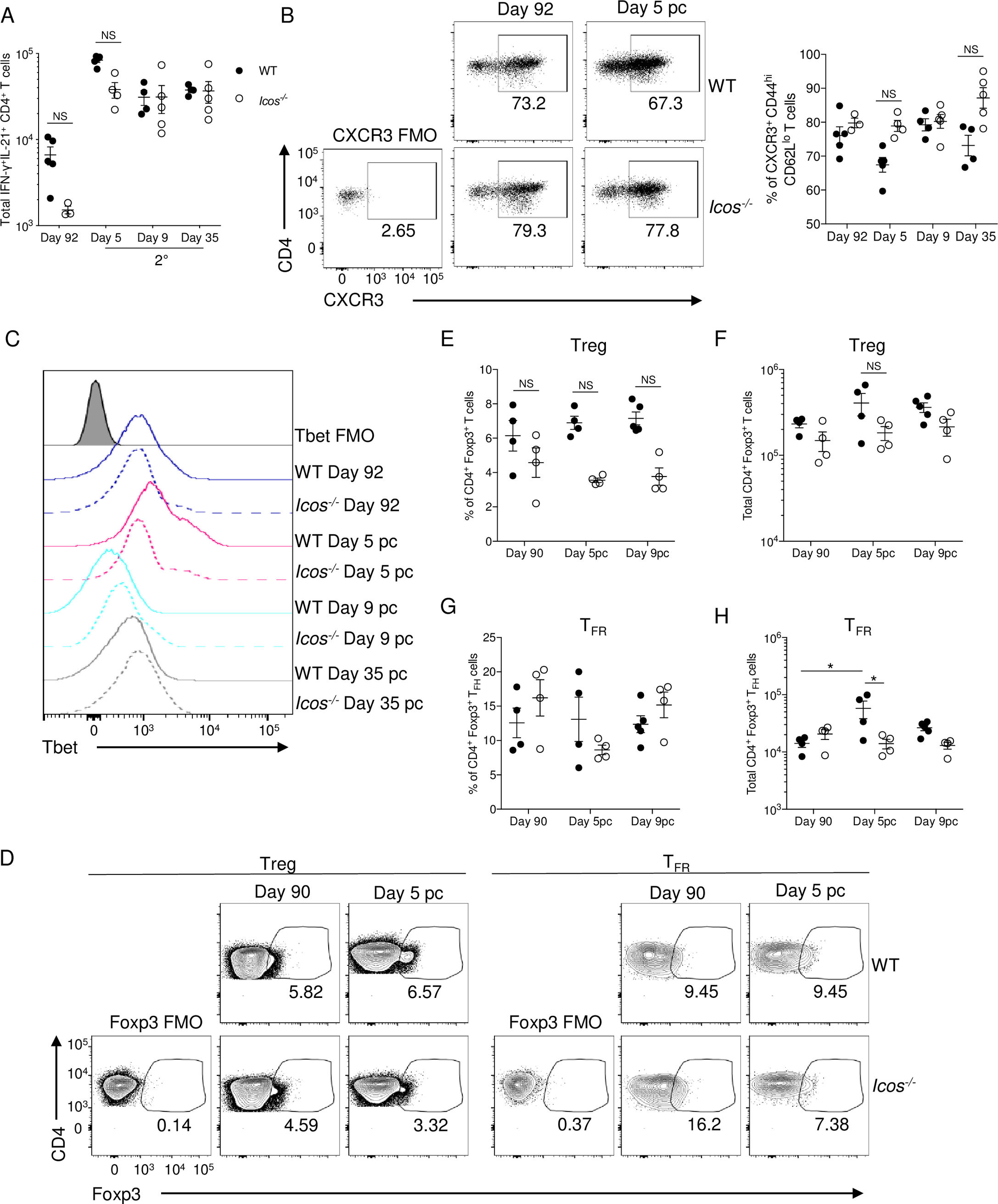

Supplement: S2 Fig — (A) Total IFN-γ +IL-21+ CD4+ T cells after restimulation of splenocytes with PMA and Ionomycin in the presence of Brefeldin A. (B) Representative flow plots and frequency of CXCR3-expressing activated CD4+ T cells. Splenocytes were gated through live singlets, and non-T lymphocytes were excluded before gating on CD4+ T cells. (C) Histogram of Tbet+ expression by CD4+ T cells at the indicated time point. (D) Representative flow plots of Foxp3+ Tregs and Tfr cells. T cells were gated through live singlets, and non-T lymphocytes were excluded before gating on CD4+ T cells. CD4+ T cells were then subgated for Foxp3 expression (Tregs) or on CXCR5+PD-1+ CD4+ T cells prior to gating on Foxp3+ cells (Tfr). Frequency (E) and total number (F) of Tregs. Frequency (G) and total number (H) of Tfr cells. (A-C) Data are representative of two experiments with at least three mice per group. (D-H) Data are from one experiment with four mice per group (error bars, s.e.m.). An aligned rank transformation was performed on non-parametric data before determining significance by two-way ANOVA with a post hoc Holm-Sidak’s multiple comparisons test. * p < 0.05, NS not significant. (TIF) [file ppat.1008527.s002.tif]

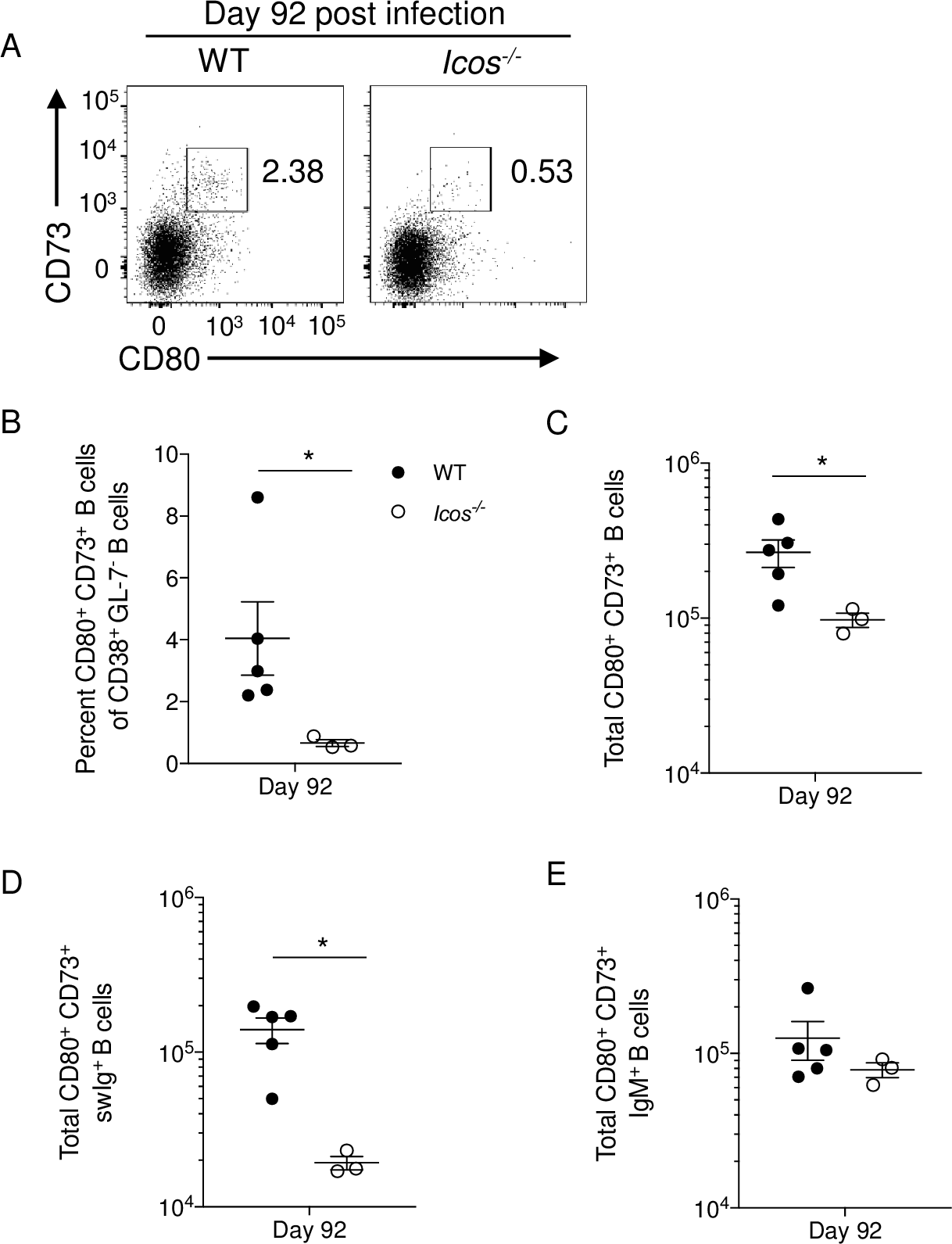

Supplement: S3 Fig — (A) Representative dot plots of CD80 and CD73 expression on live CD38+GL-7-CD19+B220+ B cells from WT and Icos-/- mice derived from the parent gate in Fig 5. Box represents CD80+CD73+ MBCs. (B) Frequency and total number (C) of CD80+CD73+ MBCs per spleen at day 92 p.i. Total number of (D) swIg+ and (E) IgM+ CD80+CD73+ MBCs per spleen at day 92. Data are representative of two independent experiments with at least three mice per group (error bars, s.e.m.). Significance was determined by a Mann-Whitney t-test. * p < 0.05. (TIF) [file ppat.1008527.s003.tif]

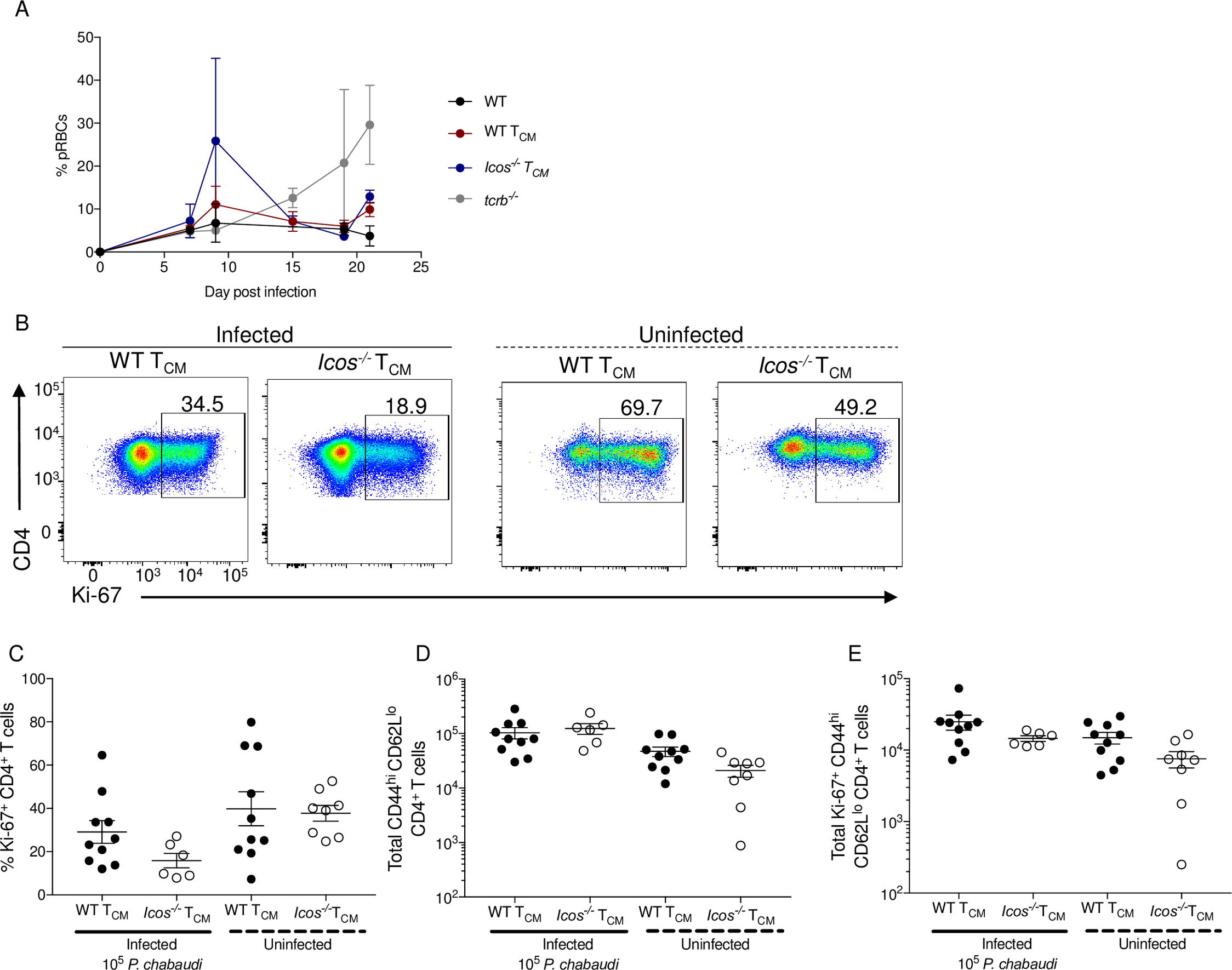

Supplement: S4 Fig — Mice were treated as in Fig 6. (A) Parasitemia curve as determined by flow cytometry. (B) Representative flow plots of live recovered CD4+CD45.1+ donor T cells expressing Ki-67. The frequency (C) of Ki-67+ CD4+CD45.1+ T cells on day 21 p.i. Total number of activated (CD44hiCD62Llo) CD45.1+CD4+ T cells (D) and Ki-67+ activated T cells (E) recovered from tcrb-/- recipient mice on day 21. Data are pooled from two independent experiments with at least three mice per group (error bars, s.e.m.). (TIF) [file ppat.1008527.s004.tif]

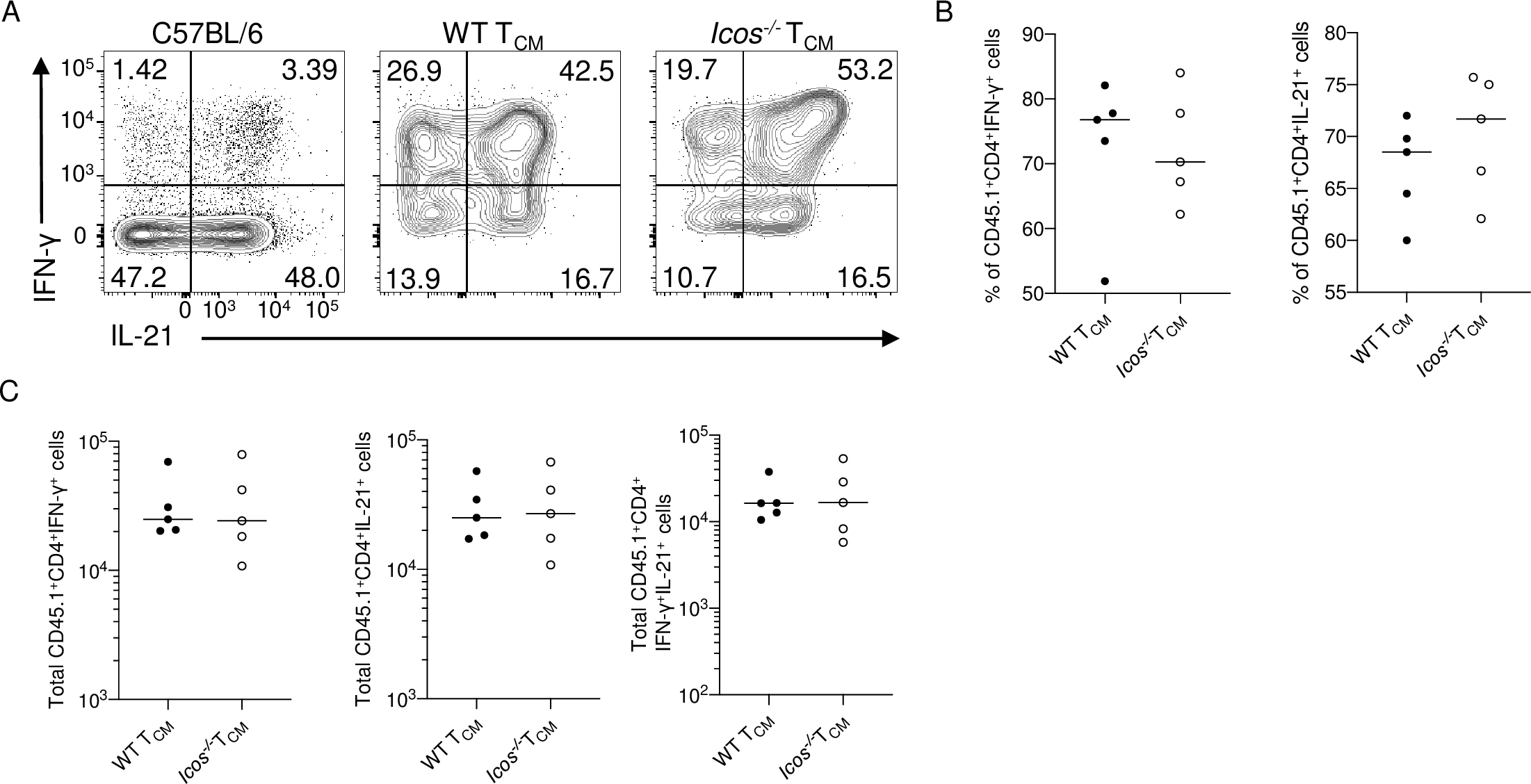

Supplement: S5 Fig — (A) Representative flow plots of IFN-γ and IL-21-expressing CD45.1+CD4+ T cells after stimulation with PMA and Ionomycin in the presence of Brefeldin A. Frequency of (B) IFN-γ+ and IL-21+ CD45.1+CD4+ T cells. Total number of (C) IFN-γ+, IL-21+, and IFN-γ+IL-21+ CD45.1+CD4+ T cells. Data are from one experiment with five mice per group (error bars, s.e.m.). Significance was determined by a Mann-Whitney t-test. (TIF) [file ppat.1008527.s005.tif]

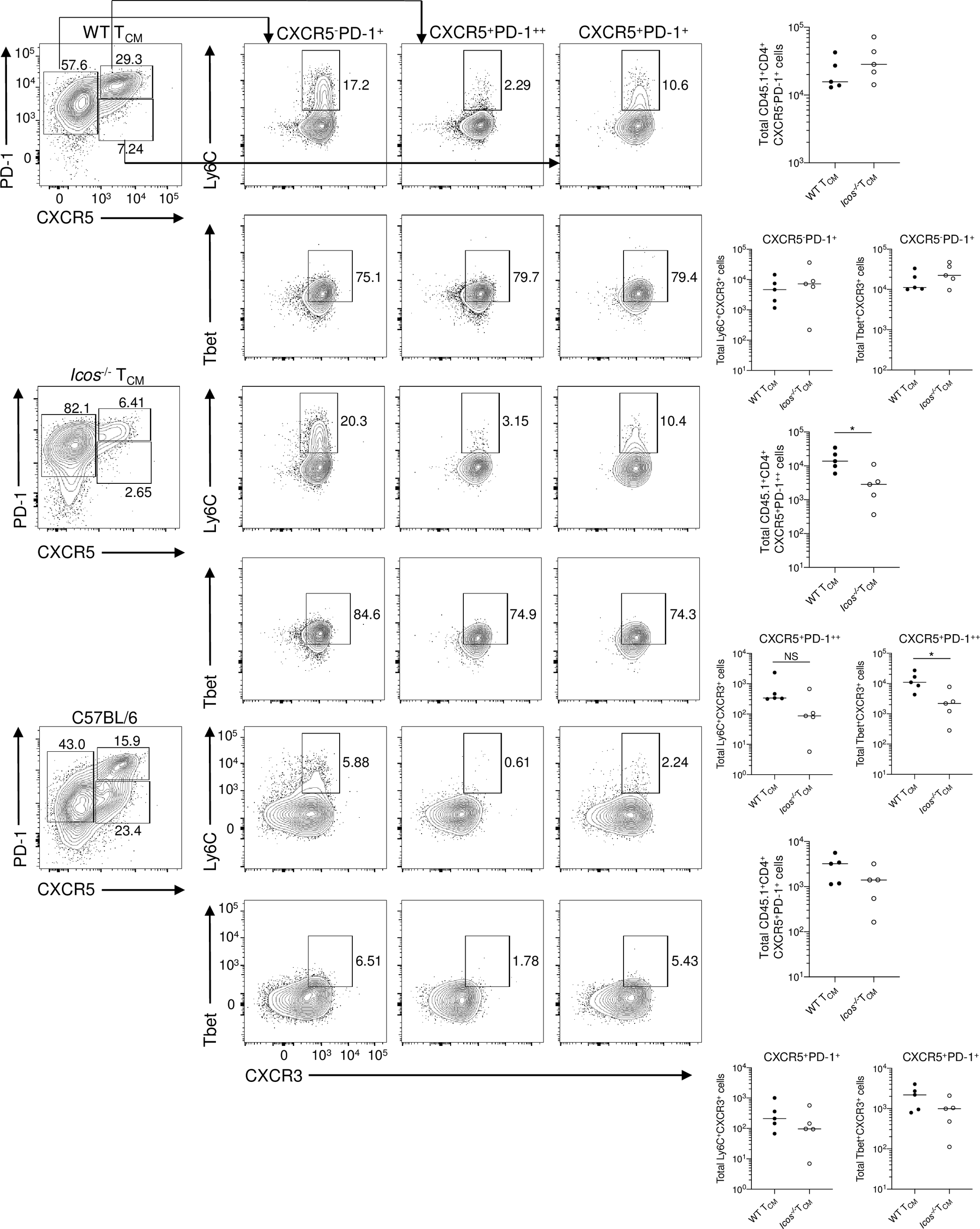

Supplement: S6 Fig — WT and Icos-/- CD45.1+CD4+ T cells recovered from tcrb-/- mice on day 21 p.i. were separated into three different gates based on their expression of PD-1 and CXCR5: PD-1+CXCR5-, Tfh-like (CXCR5+PD-1+), and GC Tfh (CXCR5+PD-1++). The three gated populations of T cells were analyzed for Ly6C, CXCR3, and Tbet expression. Graphs represent total numbers of cells for each of the subgated populations of cells. Data are from one experiment with five mice per group (error bars, s.e.m.). Significance was determined by a Mann-Whitney t-test. * p < 0.05, NS not significant. (TIF) [file ppat.1008527.s006.tif]

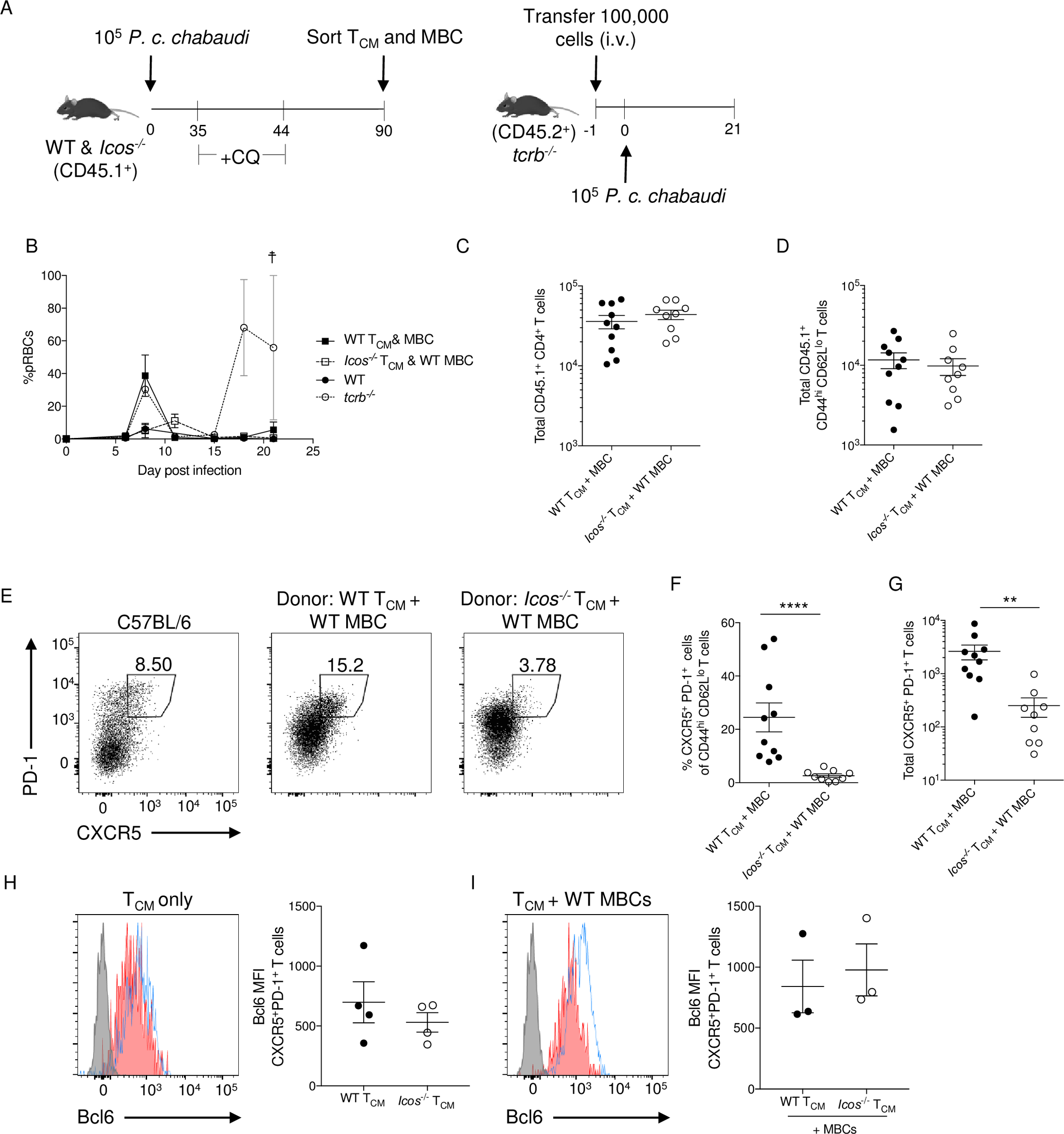

Supplement: S7 Fig — (A) Experimental model. WT and Icos-/- CD45.1+ mice were infected with 105 P. c. chabaudi pRBCs and given CQ beginning at day 35 p.i. TCM cells were sorted from WT and Icos-/- CD45.1+ mice on day 90 along with CD73+CD38+GL-7- MBCs from WT CD45.1+ mice. 100,000 cells of each TCM cell population were transferred together with an equal number of MBCs retro-orbitally into CD45.2+ tcrb-/- mice. WT CD45.2+ and tcrb-/- mice that did not receive donor cells served as controls. Twenty-four hours later, mice were infected with 105 P. c. chabaudi pRBCs. Mice were sacrificed at day 21 p.i. (B) Parasitemia curve determined by Giemsa stained thin blood smears. Cross denotes the removal of a morbid mouse from the study. Total number of live (C) and activated (CD44hiCD62Llo) CD45.1+CD4+ T cells (D) recovered from recipient mice on day 21. (E) Representative dot plots of CXCR5 and PD-1 expression on live activated CD45.1+CD4+ T cells at day 21. Polygon identifies the CXCR5+PD-1+ expressing CD4+ T cells. The frequency (F) and total number (G) of live activated CD45.1+CD4+ CXCR5+PD-1+ T cells. Representative histograms and MFI (median) of Bcl6 expression at day 21 p.i. by recovered CXCR5+PD-1+CD45.1+CD4+ T cells derived from WT (red peak) or Icos-/- (blue peak) donor (H) TCM only and (I) TCM and MBCs groups. Bcl6 FMO (gray peak). Data are pooled from three independent experiments (error bars, s.e.m.). Significance calculated by one-way ANOVA Kruskal-Wallis test with post hoc Dunn’s multiple comparisons test. ** p < 0.01, **** p ≤ 0.0001. (TIF) [file ppat.1008527.s007.tif]

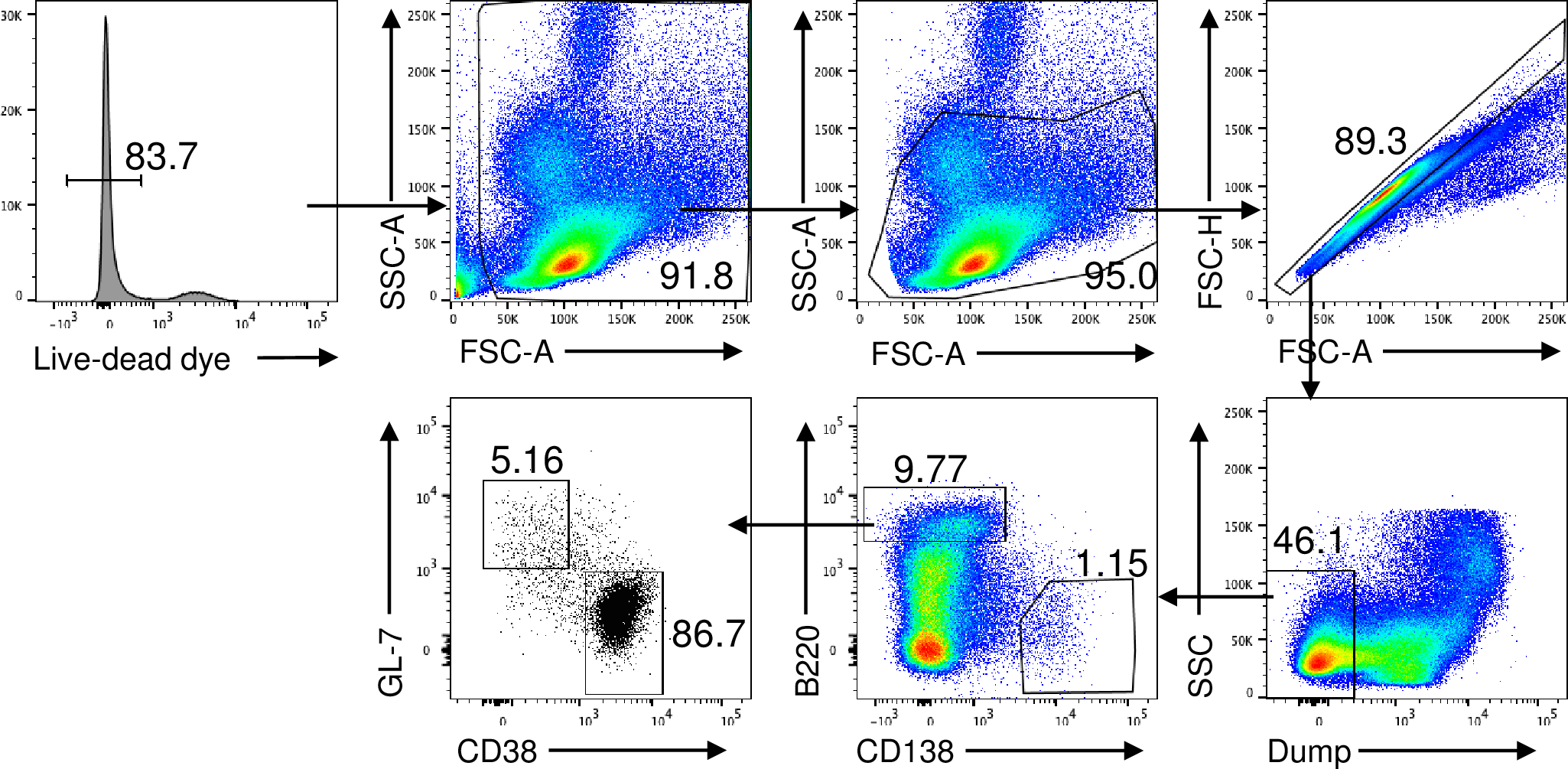

Supplement: S8 Fig — To determine the phenotype of endogenous CD45.2+ B cells, splenocytes were gated through live lymphocytes, single cells, dump- (CD3-CD11b-CD11c-Ter119-), and subsequently gated on B220+CD138- B cells or B220-CD138+ plasmablasts before reaching the gates displayed in Fig 8. (TIFF) [file ppat.1008527.s008.tiff]
